# *Mycobacterium tuberculosis* has diminished capacity to counteract redox stress induced by elevated levels of endogenous superoxide

**DOI:** 10.1016/j.freeradbiomed.2015.03.008

**Published:** 2015-07

**Authors:** Priyanka Tyagi, Allimuthu T. Dharmaraja, Ashima Bhaskar, Harinath Chakrapani, Amit Singh

**Affiliations:** a Department of Microbiology and Cell Biology, Centre for Infectious Disease Research (CIDR), Indian Institute of Science, Bangalore-12, India; b International Centre for Genetic Engineering and Biotechnology, New Delhi 67, India; c Department of Chemistry, Indian Institute of Science Education and Research, Pune 08, India

**Keywords:** CFZ, clofazimine, DHE, dihydroethidium, MABA, microplate Alamar blue assay, MDR, multidrug-resistant, *Msm*, *Mycobacterium smegmatis*, *Mtb*, *Mycobacterium tuberculosis*, XDR, extensively drug-resistant, Drug resistance, *Mycobacterium smegmatis*, *Mycobacterium tuberculosis*, Reactive oxygen species (ROS), Redox regulation, Superoxide

## Abstract

*Mycobacterium tuberculosis* (*Mtb*) has evolved protective and detoxification mechanisms to maintain cytoplasmic redox balance in response to exogenous oxidative stress encountered inside host phagocytes. In contrast, little is known about the dynamic response of this pathogen to endogenous oxidative stress generated within *Mtb*. Using a noninvasive and specific biosensor of cytoplasmic redox state of *Mtb*, we for first time discovered a surprisingly high sensitivity of this pathogen to perturbation in redox homeostasis induced by elevated endogenous reactive oxygen species (ROS). We synthesized a series of hydroquinone-based small molecule ROS generators and found that ATD-3169 permeated mycobacteria to reliably enhance endogenous ROS including superoxide radicals. When *Mtb* strains including multidrug-resistant (MDR) and extensively drug-resistant (XDR) patient isolates were exposed to this compound, a dose-dependent, long-lasting, and irreversible oxidative shift in intramycobacterial redox potential was detected. Dynamic redox potential measurements revealed that *Mtb* had diminished capacity to restore cytoplasmic redox balance in comparison with *Mycobacterium smegmatis* (*Msm*), a fast growing nonpathogenic mycobacterial species. Accordingly, *Mtb* strains were extremely susceptible to inhibition by ATD-3169 but not *Msm*, suggesting a functional linkage between dynamic redox changes and survival. Microarray analysis showed major realignment of pathways involved in redox homeostasis, central metabolism, DNA repair, and cell wall lipid biosynthesis in response to ATD-3169, all consistent with enhanced endogenous ROS contributing to lethality induced by this compound. This work provides empirical evidence that the cytoplasmic redox poise of *Mtb* is uniquely sensitive to manipulation in steady-state endogenous ROS levels, thus revealing the importance of targeting intramycobacterial redox metabolism for controlling TB infection.

## Highlights

• Dynamic response of *Mycobacterium tuberculosis* (*Mtb*) to endogenous ROS is poorly characterized.
• Biochemically and genetically validated ROS generators were used to enhance ROS in *Mtb*.
• Application of a cytoplasmic redox biosensor revealed dynamic changes in redox potential of *Mtb* on elevated endogenous ROS.
• *Mtb* has diminished capacity to restore redox balance in response to endogenous ROS.
• Maintenance of cytoplasmic redox potential in drug-resistant *Mtb* is crucial for its survival.

## Graphical abstract

**Figure:**
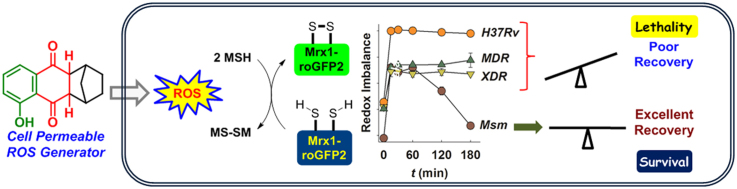

## Introduction

Reactive oxygen species (ROS) and reactive nitrogen species (RNS) produced by the host enzymes are critical for controlling *Mycobacterium tuberculosis*, the causative agent of tuberculosis [1–3]. Moreover, both clinical observations in patients suffering from chronic granulomatous disease and recent experimental studies in zebra fish indicate a major role of superoxide (O_2_^−•^) generated by NOX2 in neutrophil-mediated containment of mycobacterial infection *in vivo* [4,5]. Collectively, these studies suggest a beneficial role of host-generated ROS and RNS in limiting survival of *Mtb* during infection. Despite the toxic effects of ROS and RNS, *Mtb* survives and persists within macrophages, indicating that *Mtb* has defense mechanisms to effectively counter host-generated exogenous oxidative stress. In line with this, several studies have reported the essential role of cell wall-associated lipids (cycloproponated mycolic acids, PDIM, etc.) [6,7], secretory antioxidant enzymes (superoxide dismutase [SodA, SodC], catalase [KatG], etc.), and secretory redox buffer ergothionine (ERG) in providing an excellent anatomical barrier to and detoxification of exogenous oxidants [8]. Additionally, *Mtb* produces cytosolic reducing buffers such as mycothiol (MSH) and thioredoxins (Trxs) to protect the cytoplasmic redox environment from oxidative insult. However, while lack of antioxidant secretion and cell wall lipid components adversely affect survival of *Mtb in vivo* [9,10], disruption of the cytoplasmic redox systems MSH or Trxs does not attenuate *Mtb* replication *in vivo* [11]. These findings indicate that cell surface-associated mechanisms (e.g., SodA, KatG, mycolic acids) are necessary and sufficient to alleviate toxicity mediated by exogenous ROS and these mycobacterial adaptive mechanisms seem to be important for maintaining intrabacterial redox balance and persistence of *Mtb* during infection. A modest increase in the endogenous O_2_^−•^ levels is known to substantially damage DNA, proteins, and lipids in diverse bacteria [12,13], indicating that in the case of *Mtb* infection, host-generated O_2_^−•^ are effectively countered by mycobacterial protective systems to prevent overwhelming amplification of endogenous oxidants in *Mtb* which exceeds the organism׳s detoxification and repair capabilities. To gain a direct insight into this mechanism, we have recently developed an elegant tool (Mrx1–roGFP2) to precisely measure dynamic changes in the mycothiol redox potential (*E*_*MSH*_) of *Mtb* during infection. Using this bioprobe, we demonstrated transient oxidative changes in the *E*_*MSH*_ of *Mtb* inside macrophages [14], which concurs with the natural tolerance of *Mtb* toward oxidative stress in phagocytes. Together, these findings indicate that *Mtb* is genetically evolved to dissipate extracellular oxidative stress encountered during infection.

Because of the robust mechanisms mitigating extracellular oxidative stress, how *Mtb* responds to specific elevation in endogenous ROS levels has never been studied. Intriguingly, recent studies indirectly indicate that *Mtb* might be sensitive to increases in endogenous ROS levels. For example, rifampicin, a known RNA polymerase inhibitor, was found to enhance ROS including hydroxyl radicals in *Mtb* [15,16]. However, the mechanism by which rifampicin generate ROS is poorly understood and thus its lethality cannot be attributed to ROS alone. Similarly, clofazimine has been shown to act through induction of oxidative stress due to elevated ROS [17]. Due to its ability to intercalate DNA [18], this drug is not a reliable prototype to study the specific effects of elevated ROS. Although the antimalarial drug artemisinin that acts through induction of oxidative stress is itself not a *Mtb* inhibitor, it has recently been reported that when covalently linked with mycobactin, a siderophore which enhances cell permeability, the resulting conjugate is a potent antimycobacterial agent [15]. However, the requirement for iron as a trigger for oxidative stress induction by artemisinin as well as the presence of the iron-chelating mycobactin complicates mechanistic interpretations regarding the mycobactericidal role of ROS. Together, while these studies partly provide evidence for the sensitivity of *Mtb* toward oxidative stress, a clear understanding of how pathogens respond to endogenous ROS generated within *Mtb* cells remained uncharacterized.

In order to better understand the effects of elevated ROS in *Mtb*, we decided to test the consequences of exposure of *Mtb* to a set of novel, cell-permeable, reliable, and efficient superoxide radical generators (ATD series). Using multiple experimental approaches involving sensitive chemical, enzymatic, genetic, and expression assays, we investigated the potential of ATD compounds in (i) enhancing endogenous mycobacterial ROS production and (ii) impairing intramycobacterial redox homeostasis. The exceptional growth inhibitory activity shown by ATD compounds against drug-susceptible and drug-resistant strains of *Mtb* unambiguously demonstrates that intramycobacterial redox state is exceptionally sensitive to endogenous oxidative stress. Our mechanistic findings open up fresh avenues of redox research pertaining to the development of ROS generating anti-TB drugs.

## Materials and methods

### Dihydroethidium assay for superoxide detection

Dihydroethidium (DHE) assay was used for superoxide detection [19–21]. A stock solution of dihydroethidium (0.93 mg, 2.95 μmol) was prepared in DMSO (295 μl) and stored in the dark at −20 °C until its use. A stock solution of the compound (5 μl, 10 mM) and DHE (5 μl of 10 mM) were reacted in acetonitrile:phosphate buffer of pH 7.4 (1:1, v/v, 100 mM, final volume 100 μl) for 3 h at 37 °C. The reaction mixture was diluted to 50 μM using acetonitrile:phosphate buffer of pH 7.4 (1:1, v/v, to a final volume of 1 ml). The reaction mixture was filtered (0.22 μm) and injected (25 μl) in an Agilent high performance liquid chromatograph (HPLC) attached with a fluorescence detector (excitation at 356 nm; emission at 590 nm). The column used was a Zorbax SB C-18 reversed-phase column (250×4.6 mm, 5 μm), the mobile phase was water:acetonitrile containing 0.1% trifluoroacetic acid and a gradient starting with 90: 10%→0 min, 90: 10 to 70: 30→0–45 min, 30:70 to 0: 100→45–50 min, 0: 100→50–55 min, 10: 90→55–60 min was used with a flow rate of 0.5 ml/min. Under these conditions literature reports indicate that if superoxide is produced, a peak for 2-hydroxyethidium (2-OH-E^+^), which elutes at 28.6 min, would be observed [19–21]. When other oxidative species are generated ethidium (E^+^) would be formed. Again, according to literature reports E^+^ elutes at 29.5 min [19–21]. We independently confirmed the formation of 2-OH-E^+^ and E^+^ by mass spectrometry.

### Intracellular superoxide detection

For intracellular superoxide detection [19–21], *Mycobacterium smegmatis* (MC^2^155) was cultured in 5 ml of middle brook M7H9 medium (M7H9, with 10% albumin–dextrose–saline (ADS) supplement) at 37 °C for 24 h. The cultured bacteria were centrifuged to aspirate out the medium and resuspended to an OD _600_ _nm_ of 0.5 with fresh M7H9 medium. This bacterial solution was incubated with 50 μM of each ATD compound and 50 μM dihydroethidium independently for 30 min in the dark by covering the falcon tube in an aluminum foil. The suspension was centrifuged to aspirate out any excess of the compound and/or DHE in the medium. The collected bacterial pellet was resuspended with acetonitrile and the cells were lysed using a probe sonicator for 3 min on ice. The cell lysate was then removed by centrifugation and the supernatant acetonitrile was separated and stored at −20 °C before injecting in HPLC. The HPLC method used was as described previously [19–21]. A similar protocol was followed for the detection of intracellular superoxide generation by ATD-3169 in *Mycobacterium bovis BCG*.

### Preparation of mycobacterial cells for E_MSH_ measurements by flow cytometry

Mycobacterial strains were grown in Middlebrook 7H9 medium (Difco) supplemented with 10% ADS, 0.2% glycerol, 0.05% Tween 80 treated with 10 mM NEM for 5 min at room temperature (RT) followed by fixation with 4% PFA for 15 min at RT. After washing thrice with 1X phosphate buffer saline (PBS), bacilli were analyzed using a BD FACS Verse Flow cytometer (BD Biosciences). The biosensor response was measured by analyzing the ratio at a fixed emission (510 nm) after excitation at 405 and 488 nm. Data were analyzed using the FACSuite software.

### MIC assays

MIC was determined using a microplate Alamar blue assay (MABA). The Alamar blue assay was performed in 96-well flat bottom plates. *Mtb* strains were cultured in 7H9-ADS medium and grown till exponential phase (OD _600_ _nm_ ~0.4). Approximately 1×10^5^ bacteria were taken per well in a total volume of 200 µl of 7H9-ADS medium. Wells containing no *Mtb* were the autofluorescence control. Additional controls consisted of wells containing cells and medium only. After 5 days of incubation at 37 °C, 20 µl of 10X Alamar blue was added and plates were reincubated for 24 h. The fluorescence readings were then recorded. Fluorescence intensity was measured in a SpectraMax M3 plate reader (Molecular Device) in top-reading mode with excitation at 530 nm and emission at 590 nm. Percentage inhibition was calculated based on the relative fluorescence units and the minimum concentration that resulted in at least 90% inhibition was identified as MIC.

### Checkerboard synergy assay

Checkerboard synergy assay was performed using Alamar blue dye in a 96-well plate as described [22]. Synergy was defined by fractional inhibitory concentration index (FICI) values of 0.5, antagonism by FICI values of >=4.0, and no interaction by FICI values from 0.5 to 4.0. The ΣFICs were calculated as follows: ΣFICI=FIC A+FIC B, where FIC A is the MIC of drug A in the combination/MIC of drug A alone, and FIC B is the MIC of drug B in the combination/MIC of drug B alone.

Other assay protocols, synthetic procedures, and characterization data are available in the Supplemental Information.

## Results

### 2,3-Dihydro-1,4,-naphthooquinone-based small molecules generate ROS inside mycobacteria

**Fig. 1:**
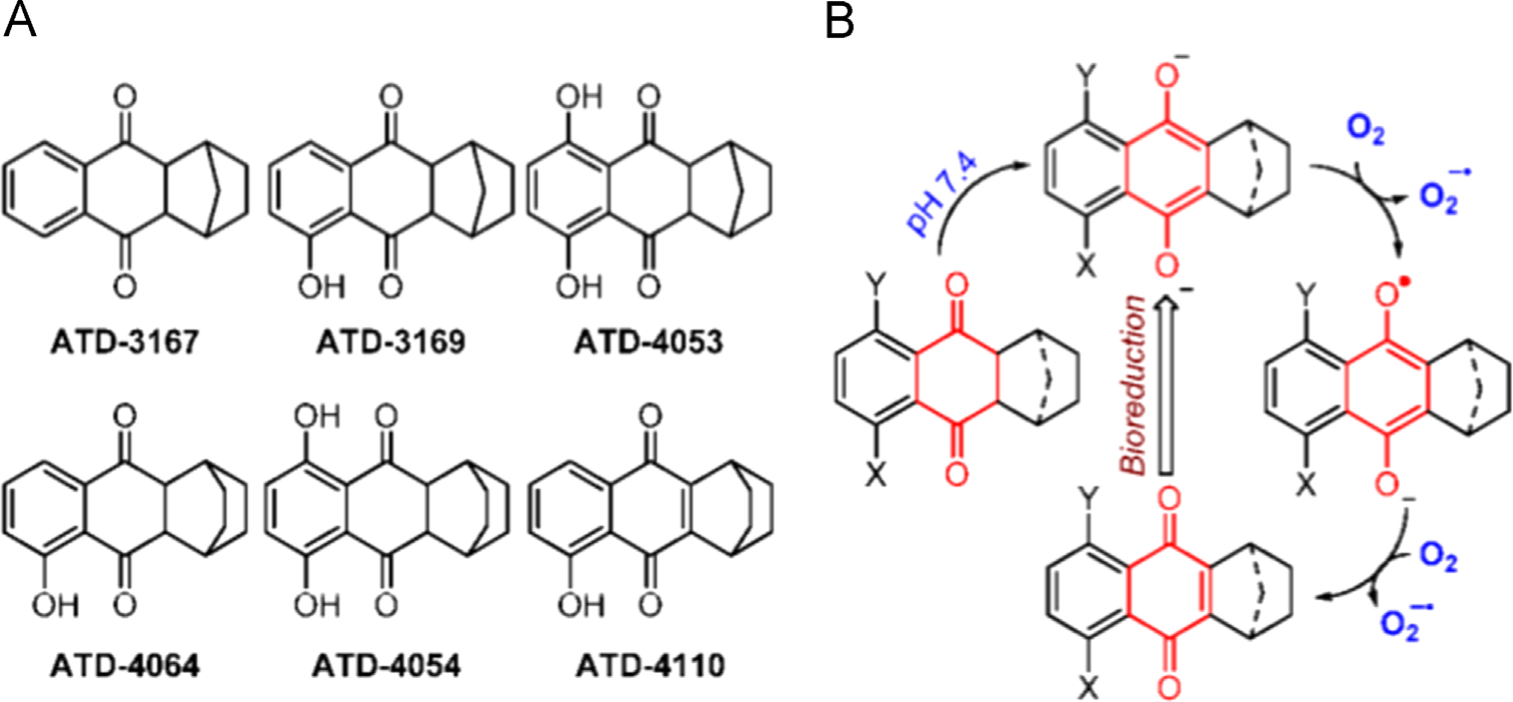
(A) Structures of ATD series of compounds prepared in this study. (B) Mechanism of O_2_^−•^ radical generation from 2,3-dihydro-1,4,-naphthoquinones involves enolization in buffer to a diolate, which spontaneously produces superoxide by reacting with oxygen to give a quinone which in turn is known to undergo bioreduction to generate superoxide.

To examine the response of *Mtb* toward endogenous oxidative stress, our first challenge was to synthesize cell-permeable redox-oriented compounds that specifically enhance ROS levels within *Mtb* cells. Our preliminary analysis recently demonstrated growth inhibitory activity of ROS generators based on a 2,3-dihydro-1,4,-naphthoquinone scaffold against mycobacteria [23]. While these molecules are believed to generate intrabacterial ROS [23], a direct proof for this and the exact mechanism by which they perturb mycobacterial redox physiology to exert antimycobacterial activity remain uncharacterized. To investigate this, we first synthesized a new series of ROS-generating compounds (ATD series) in 80–90% yield (Fig. 1A and Table S1) and characterized by NMR (see SI Experimental procedures). These compounds undergo enolization in buffer to produce a hydroquinone which is known to react with oxygen to generate up to 2 mol of superoxide (Fig. 1B) [24]. Furthermore, after generation of ROS, a naphthoquinone would be produced, which in turn can produce ROS through redox cycling (Fig. 1B). Thus, we predict that an ATD series of compounds can potentially target mycobacterium redox homeostasis through (i) spontaneous generation of ROS by directly reducing oxygen, and (ii) elevating intramycobacterial ROS levels by undergoing redox cycling inside mycobacteria.

**Fig. 2:**
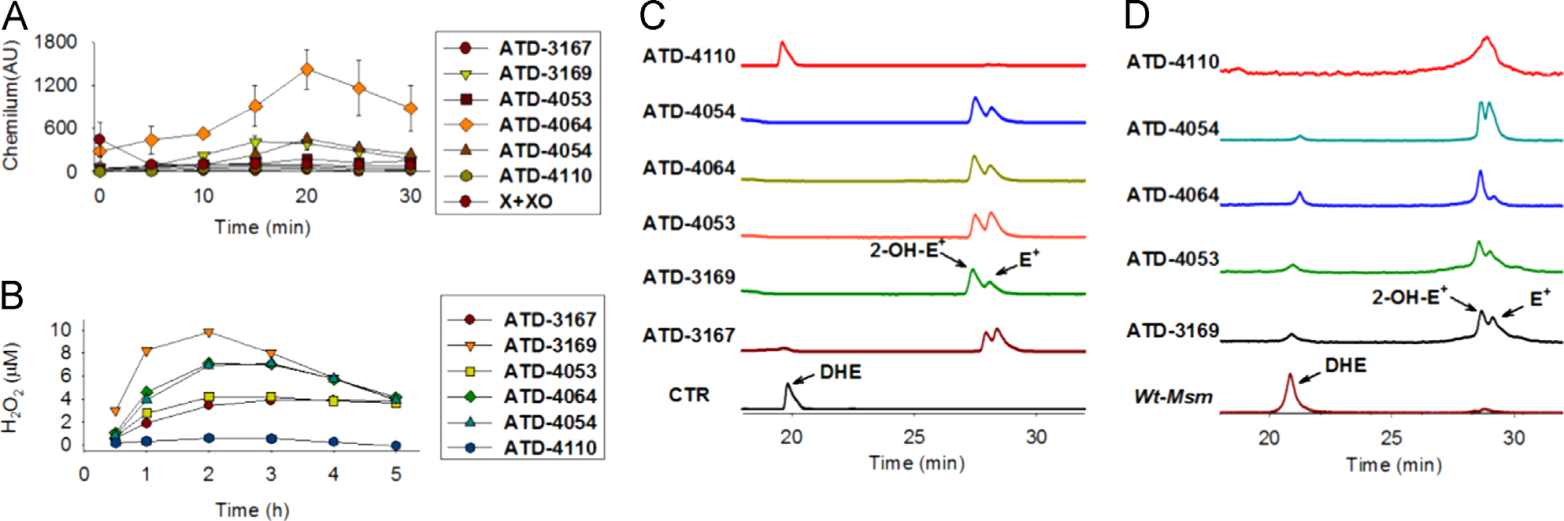
(A) The time course of superoxide radical anion (O_2_^−•^) generation during incubation in pH 8.0 buffer over 30 min was studied by a luminol chemiluminescence assay. (Note: X+XO generates O_2_^−•^ rapidly, typically <1 min). (B) Time course of hydrogen peroxide (H_2_O_2_) generated during incubation of *Wt*-*Msm* with 10 µM of the compound in pH 7.4 was estimated using an Amplex Red fluorescence assay. (C) A HPLC-based dihydroethidium (DHE) assay was used to infer generation of O_2_^−•^ after incubation of compounds (50 µM) in pH 8.0 buffer for 1 h. 2-Hydroxyethidium (2-OH-E^+^), which is exclusively formed by the reaction of O_2_^−•^ with DHE, elutes at 28.6, and ethidium E^+^, which is formed by nonspecific oxidation of DHE, elutes at 29.5 min. (D) HPLC traces of assay for intracellular O_2_^−•^production using a dihydroethidine (DHE) assay in *Mycobacterium smegmatis* (*Wt*-*Msm*). Incubation with compounds (50 µM) was for 30 min and DHE levels indicate unoxidized dye while 2-OH-E^+^ formed is an indicator for O_2_^−•^ production and E^+^ is indicative of increase in oxidative species.

To examine both possibilities, we first monitored the ability of ATD compounds to generate O_2_^−•^ under ambient aerobic conditions in buffer adjusted to pH 8.0 using a widely reported luminol-based chemiluminescence assay [25]. Xanthine oxidase (XO), an enzyme which oxidizes hypoxanthine (X) to produce O_2_^−•^ and other oxidants, served as a positive control in our assays. As a negative control, we have synthesized a closely related derivative of ATD compounds (ATD-4110), which is a naphthoquinone and is therefore incapable of spontaneously producing O_2_^−•^ in buffer. The compounds ATD-3169, ATD-4064, and ATD-4054 produced significantly higher levels of O_2_^−•^ as revealed by ~2- to 15-fold increased chemiluminescence as compared to positive control at 20 min postincubation (Fig. 2A; also see Supplemental Information Fig. S1). A time-dependent analysis demonstrated an initial increase in O_2_^−•^ production for 20 min followed by a gradual decline (Fig. 2A). Since the main aim of this work is to induce intramycobacterial O_2_^−•^ generation by ATD compounds, we determined O_2_^−•^ production using a well-established and freely cell-permeable O_2_^−•^ indicator, dihydroethidium [26]. It has been shown that DHE specifically reacts with O_2_^−•^ to release fluorescent product 2-hydroxyethidium (2-OH-E^+^), which can be conveniently detected by analytical techniques such as HPLC, fluorescence microscopy, etc. [26]. The reaction of DHE with other oxidants produces ethidium (E^+^). We first confirmed O_2_^−•^ generation in the buffer at pH 8.0 using DHE. An HPLC spectrum of O_2_^−•^ reaction products clearly showed varying levels of 2-OH-E^+^ and E^+^ generated by ROS generators (Fig. 2C). As expected, peaks correspond to 2-OH-E^+^ and E^+^ were not detected in buffer alone or in the case of compound ATD-4110 (Fig. 2C).

To evaluate if our ROS generators are capable of promoting O_2_^−•^ inside mycobacterial cells, we exposed *Mycobacterium smegmatis* to ATD compounds and DHE and detected fluorescent peaks by HPLC. Since cell death induced by ATD compounds might also elevate intracellular ROS levels, O_2_^−•^ generation was monitored at an early time point (60 min). Further, to minimize the contribution of extracellular O_2_^−•^ present in the culture medium, we thoroughly washed components of 7H9 growth medium and performed measurements on cells suspended in 1X PBS. As shown in Fig. 2D, intramycobacterial O_2_^−•^ was generated by all of the ATD compounds. Interestingly, ATD-4110 generated comparable levels of intramycobacterial O_2_^−•^, thus validating the redox-cycling-based mechanism of ROS production within *Msm* cells by these compounds. Exogenous O_2_^−•^ cannot penetrate the cell membrane [27], suggesting that ATD compounds are permeating into the cell interior and promoting endogenous O_2_^−•^ production possibly by catalyzing electron cycling with flavin, Fe–S clusters, and oxygen [28]. In a separate experiment, *Msm* cells pretreated with DHE were then exposed to ATD-3169 and we similarly found an increase in 2-OH-E^+^, supporting the capability of this compound to permeate mycobacteria to enhance intracellular ROS levels (see Supplemental Information Fig. S2).

Since O_2_^−•^ rapidly undergoes dismutation to H_2_O_2_ and molecular oxygen (O_2_) either spontaneously or by superoxide dismutase (SOD) [28], we next analyzed the ability of ATD compounds to generate H_2_O_2_. We incubated *Msm* with 50 μM of compounds for 60 min and H_2_O_2_ released in the extracellular medium was measured using a well-established fluorescence-based Amplex Red assay [29]. A calibration curve was generated with varying concentrations of H_2_O_2_ and the levels of extracellular H_2_O_2_ can be quantified by this assay (Fig. S1). We observed that while all of the compounds produce H_2_O_2_, ATD-3169 showed exceedingly greater levels of H_2_O_2_ in 2 h (Fig. 2B). Furthermore, consistent with the O_2_^−•^ data, time–kinetic analysis demonstrated an initial increase in H_2_O_2_ production reaching a maximum value at 2 h posttreatment, followed by a steady decrease (Fig. 2B). The eventual decrease in O_2_^−•^ and H_2_O_2_ levels is in line with our recent study demonstrating the presence of an efficient antioxidant buffer system (mycothiol; MSH) to rapidly counterbalance oxidative stress in *Msm* [14]. Taken together, we have successfully designed novel small molecules capable of enhancing intracellular ROS in mycobacteria.

### ROS generators modulate mycothiol redox balance in Msm

We have shown the generation of O_2_^−•^ in buffer and inside *Msm* by ATD compounds using redox-active fluorogenic dyes and HPLC. Although important for detecting ROS *in vitro*, intracellular detection of ROS by fluorogenic dye-based disruptive technologies can introduce oxidation artifacts during cell lysis, which prohibits dynamic measurements in real time [30,31]. Therefore, to validate our *in vitro* findings and to find out if ATD compounds are efficient in overwhelming mycobacterial antioxidant capacity, we next exploited a recently reported noninvasive tool to measure dynamic changes in intramycobacterial oxidation–reduction state [14]. Actinomycetes including mycobacteria produce millimolar concentrations of an antioxidant, mycothiol, which serves as a major cytoplasmic buffer that protects bacteria from oxidative stress [32]. Since oxidative stress rapidly oxidizes reduced mycothiol (MSH) to oxidized mycothiol (MSSM), the ratio of mycothiol redox couple (MSH/MSSM) or mycothiol redox potential (*E*_*MSH*_) provides a direct indicator of mycobacterial redox state [14]. To examine if the ROS generators perturb mycobacterial redox balance, we first performed dynamic measurement *E*_*MSH*_ of *Msm* in response to ATD compounds using a highly sensitive and specific biosensor of intramycobacterial mycothiol redox potential (*E*_*MSH*_; Mrx1–roGFP2) [14]. In Mrx1–roGFP2, oxidation–reduction-sensitive GFP (roGFP2) is covalently fused to *Mtb* mycothiol-specific oxidoreductase mycoredoxin-1 (Mrx-1), which reversibly transfers electrons between the mycothiol redox buffer and the thiol groups of roGFP2 [14]. Therefore, continuous formation and release of the roGFP2 disulfide bridge are coupled with the actual redox potential of the mycothiol buffer inside mycobacteria [14]. The oxidation and reduction state of Mrx1–roGFP2 thiols can be easily detected by measuring fluorescence intensity at 405 and 488 nm excitation wavelengths with a fixed emission wavelength of 510 nm [14]. Increase or decrease in Mrx1–roGFP2 ratio (405/488) indicates oxidative or reductive shift in *E*_*MSH*_ of a mycobacterial cell, respectively [14].

**Fig. 3:**
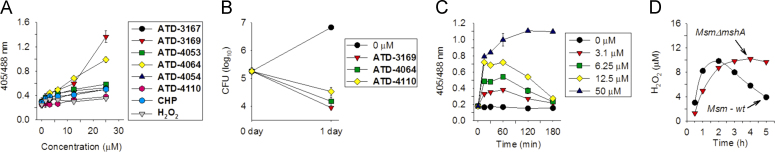
ATD compounds induce an oxidative shift in *E*_*MSH*_ of *Msm*. (A) *Msm* expressing Mrx1–roGFP2 was treated with various ATD series compounds, H_2_O_2_ and CHP (from 1.5–25 μM) for 24 h and the sensor response was measured by flow cytometry by exciting at 405 and 488 nm lasers and constant emission at 510 nm. (B) *Msm* was grown to an OD_600 nm_ of 0.6, diluted to an OD_600 nm_ of 0.06 and treated with ATD-3169 (50 µM), ATD-4064 (200 µM), ATD-4110 (200 µM) for 24 h. CFUs were plated at 0 and 24 h on 7H11 solid media supplemented with ADS. (C) *Msm* expressing Mrx1–roGFP2 was treated with nonlethal (3.1, 6.25, 12.5 μM) and lethal (50 µM) concentrations of ATD-3169, and the sensor response was measured by flow cytometry at various time points. Error bars represent standard deviations from the mean. Data shown are the representative of at least three different experiments performed in triplicate. (D) The time course of H_2_O_2_ generated during incubation of *Wt*-*Msm* and *Msm*Δ*mshA* strains with 10 µM of the ATD-3169 compound in pH 7.4 was estimated using an Amplex Red fluorescence assay.

*Msm* expressing Mrx1–roGFP2 biosensor was independently exposed to various concentrations of ATD compounds (1.5 to 25 μM) and two known ROS generators (H_2_O_2_ and cumene hydroperoxide [CHP]). The ratiometric response was measured by flow cytometry at 24 h posttreatment. Surprisingly, despite showing increased generation of ROS inside *Msm*, most of the ATD compounds were ineffective in increasing the Mrx1–roGFP2 ratio at each concentration tested, indicating an effective induction of mycothiol antioxidant system in response to ROS generators (Fig. 3A). Similar results were obtained with H_2_O_2_ and CHP treatment (Fig. 3A). Only treatment with 25 μM of ATD 3169 and ATD 4064 induced significant oxidative shift in *E*_*MSH*_ of *Msm* (Fig. 3A). To examine if enhanced oxidative *E*_*MSH*_ induced by ATD-3169 and ATD 4064 correlated with growth inhibition, we determined minimal inhibitory concentrations (MICs) of these compounds against *Msm* using MABA. Alamar blue (AB) is an oxidation–reduction indicator dye that has been widely used to measure the sensitivity of mycobacteria to anti-TB drugs [33]. A change in nonfluorescent blue to fluorescent pink indicates reduction of AB due to growth, whereas inhibition of growth by antimycobacterial compounds interferes with AB reduction and color development. Consistent with the Mrx1–roGFP2 findings, most of the ROS generators were inefficient in inhibiting growth of *Msm* even at concentrations higher than 200 μM (Table 1). Importantly, ATD-3169 completely prevented *Msm* growth at a minimum inhibitory concentration (MIC) of 50 μM, while lower oxidative stress induced by ATD-4064 correlated with the higher MIC value against *Msm* (200 μM, Table 1). It can be argued that the use of redox-cycling compounds can influence AB oxidation–reduction to compromise the MABA assay. To address this issue, we validated the inhibitory effect of a few ATD compounds (ATD 3169, ATD 4064, and ATD 4110) on *Msm* by analyzing colony forming units (CFUs). As shown in Fig. 3B, *Msm* treated with ATD compounds at MIC concentrations displayed~2.5 log reduction in CFUs as compared to untreated cells at 24 h posttreatment. These results confirm that both MABA and CFU analyses are in reasonable agreement with each other and that ATD compounds exert bactericidal effects on mycobacterial cells.

A direct linkage between oxidative *E*_*MSH*_ and growth inhibition suggests that disruption of mycothiol redox homeostasis is the underlying mechanism by which ATD-3169 exerts an antimycobacterial effect. However, ATD-3169 was ineffective in reducing *Msm* growth at concentrations lower than 25 μM. We reasoned that this could be due to efficient recruitment of the mycothiol antioxidant system to dissipate ROS generated by lower concentrations of ATD-3169. To show this, we exposed *Msm* expressing Mrx1–roGFP2 to nontoxic concentrations of ATD-3169 (3.1, 6.25, 12.5 μM) and the ratiometric response was measured at early time points to capture dynamic changes in *E*_*MSH*_. Addition of ATD-3169 resulted in a rapid increase in Mrx1–roGFP2 ratio in a concentration-dependent manner (Fig. 3C). As indicated in Fig. 3C, maximum oxidation induced by various concentrations of ATD-3169 was achieved within 15 min postexposure. Interestingly, the observed increase in Mrx1–roGFP2 ratio was not long lasting and cells recovered from the oxidative insult as indicated by a decrease in 405/480 ratio to basal levels within 180 min posttreatment (Fig. 3C), indicating activation of an MSH-dependent antioxidative mechanism. As a control, we have similarly tracked changes in *E*_*MSH*_ on treatment of *Msm* with a toxic concentration (i.e., 50 μM) of ATD-3169. In this case, *Msm* displayed a sustained and irreversible increase in 405/488 ratio, confirming the role of overwhelming mycothiol oxidation caused by higher concentrations of ATD 3169 (Fig. 3C). Lastly, to confirm the role of MSH in tolerating oxidative stress generated by ATD-3169, we measured the MIC of ATD-3169 against MSH-negative strains of *Msm* (*MsmΔmshA* and *MsmΔmshD*). As shown in Tables 1, a 2-fold lower concentration of ATD-3169 was sufficient to inhibit growth of MSH-negative strains as compared to wt *Msm*. Consistent with these findings, Amplex Red assay showed that *MsmΔmshA* consistently displayed higher levels of extracellular H_2_O_2_ as compared to wt *Msm* (Fig. 3D). These results confirmed that *Msm* responds to ATD compounds by inducing dynamic changes in MSH redox signaling and homeostasis, and suggest that efficient generation of intrabacterial ROS may induce killing by compromising reductive capabilities of mycobacteria.

### ATD-3169 perturbs mycothiol redox homeostasis in Mtb

Several studies have indicated that slow growing mycobacteria, including *Mtb*, lack protective oxidative stress responses due to the absence of a functional copy of oxidative stress regulators such as OxyR and SoxR [34]. Moreover, pathogenic and drug-resistant *Mtb* strains displayed relatively oxidized steady-state intrabacterial *E*_*MSH*_ as compared to nonpathogenic *Msm*, and exhibited exceptional sensitivity to endogenous increase in oxidant levels [14,34,35]. On this basis, we hypothesized that *Mtb* strains might show differential sensitivity toward ATD-3169 as compared to *Msm*. To examine this possibility, we expressed Mrx1–roGFP2 in the virulent laboratory strain (*Mtb* H37Rv), and two Indian patient isolates of *Mtb*, i.e., a multidrug-resistant strain (Jal-2287) and an extensively drug-resistant strain (MYC 431) [14].

**Fig. 4:**
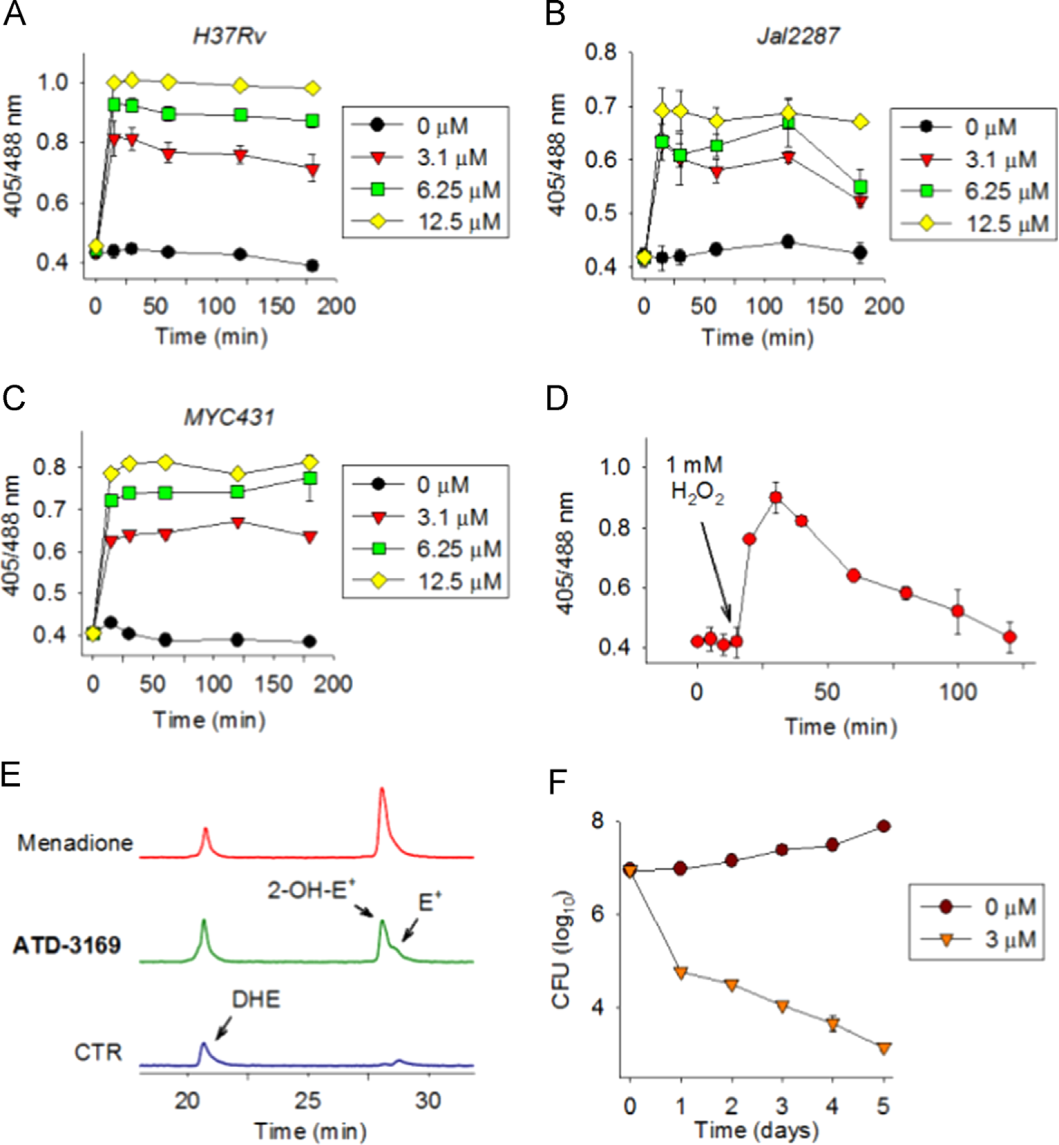
ATD-3169 induces a long-lasting oxidative shift in *E*_*MSH*_ of *Mtb* strains. *Mtb* strains (A) H37Rv, (B) Jal 2287, and (C) MYC 431 expressing Mrx1–roGFP2 were treated with ATD-3169 at various concentrations and the sensor response was measured by flow cytometry at various time points. Data shown are representative of at least three different experiments performed in triplicate. (D) H37Rv expressing Mrx1–roGFP2 was treated with 1 mM H_2_O_2_ and the sensor response was measured by flow cytometry as a function of time. Data shown are representative of at least two different experiments performed in triplicate. Error bars represent standard deviations from the mean. (E) HPLC traces of assay for intracellular O_2_^−•^ production using a dihydroethidine (DHE) assay in *BCG*. Incubation with compounds (50 µM) was for 30 min and DHE levels indicate unoxidized dye while 2-OH-E^+^ formed is an indicator for O_2_^−•^ production and E^+^ is indicative of an increase in oxidative species. Menadione is a known redox-cycling agent that enhances ROS and was used as a positive control. (F) H37Rv was grown to an OD_600 nm_ of 0.6, diluted to an OD_600 nm_ of 0.06, and treated with 3.1 μM ATD-3169 compound. At the indicated time points, CFUs were determined by plating cultures on 7H11 solid media supplemented with ADS. *P<0.05 as compared to untreated control day 0,+*P* <0.05 as compared to untreated control day 5.

*Mtb* strains expressing Mrx1–roGFP2 were exposed to lower concentrations of ATD-3169 (3.1, 6.25, 12.5 μM) at which *Msm* robustly maintained intrabacterial *E*_*MSH*_ and survival. As a positive control, we treated *Mtb* H37Rv with 1 mM H_2_O_2_. To minimize the contribution of cell death in generating oxidative stress, we measured the Mrx1–roGFP2 ratio at early time points. Addition of ATD-3169 rapidly increased the Mrx1–roGFP2 ratio in a concentration-dependent manner among the *Mtb* strains (Fig. 4A, B, and C). Similar oxidation of Mrx1–roGFP2 was detected on exogenous exposure to H_2_O_2_ (Fig. 4D). However, while the Mrx1–roGFP2 ratio gradually decreased to baseline values in the case of H_2_O_2_-challenged *Mtb* (Fig. 4D), ATD-3169-treated *Mtb* remained oxidatively stressed throughout the course of the experiment (Fig. 4A). The ratiometric fluorescence pattern displayed by the drug-resistant strains Jal 2287 and MYC 431 was comparable to that of *Mtb* H37Rv (Fig. 4B and C). Since we have shown that ATD-3169 induces an oxidative shift in *E*_*MSH*_ of *Msm* by generating endogenous ROS such as O_2_^−•^ via redox cycling, we asked if a similar mechanism exists for *Mtb*. However, due to technical and biosafety challenges associated with handling and processing a BSL3 class pathogen such as *Mtb* for HPLC analysis, we measured O_2_^−•^ levels inside a related but nonpathogenic slow growing vaccine strain of mycobacteria, *Mycobacterium bovis* BCG. It has been previously reported that the intrabacterial *E*_*MSH*_ of BCG is similar to that of *Mtb*, indicating a comparable cytoplasmic redox state of these two species under normal growing conditions [14]. Moreover, BCG was found to be equally sensitive to ATD-3169 as compared to *Mtb* strains (Table 1). Using a DHE assay, we found that BCG cells treated with ATD-3169 generate peaks corresponding to O_2_^−•^ (2-OH-E^+^) and other ROS (E^+^) (Fig. 4E). As a positive control, we used a well-known O_2_^−•^ generator (menadione) in our assay and similarly detected a 2-OH-E^+^ peak, confirming the generation and detection of O_2_^−•^ by menadione and ATD-3169 inside BCG (Fig. 4E). These results indicate that ATD-3169 severely impaired the ability of *Mtb* to orchestrate an efficient and dynamic MSH-specific antioxidant response by generating overwhelming endogenous oxidative stress.

### Low concentrations of ATD-3169 efficiently inhibit growth of drug-resistant Mtb strains

Having shown that ATD-3169 is efficient in perturbing mycothiol redox balance in *Mtb* strains, we next determined the MIC of ATD-3169 against several virulent and drug-resistant strains of *Mtb* using MABA assay. These include a laboratory strain (*Mtb* H37Rv), and several clinical drug-resistant strains of *Mtb*, i.e., single drug-resistant (BND 320), multidrug-resistant (MDR- Jal 2261, 1934, Jal 2287), and extensively drug-resistant (XDR- MYC 431) [14]. In complete agreement with the Mrx1–roGFP2 findings, virulent and drug-resistant clinical strains showed exceptional sensitivity toward ATD-3169. A concentration range of 3.125–6.25 µM was sufficient to uniformly inhibit the growth of *Mtb* strains (Table 1). The killing potential of ATD-3169 was also confirmed by enumerating colony-forming units (CFUs) of *Mtb* at various time points posttreatment (Fig. 4F). Lastly, when tested against human embryonic kidney cells HEK293, a cell viability assay revealed no significant inhibition at 25 µM [24]. Thus, at concentrations where complete inhibition of *Mtb* was observed, the compounds were well tolerated by mammalian cells. These results suggest that the endogenous production of O_2_^−•^ and H_2_O_2_ by ATD-3169 effectively perturbed mycothiol redox balance to specifically compromise growth of virulent and drug-resistant *Mtb* strains.

### Targeting mycothiol pathway increases sensitivity of Mtb to ATD-3169

Earlier studies have shown that MSH-deficient mycobacteria are susceptible to broad classes of antibiotics [36,37]. Recently we have shown that while anti-TB drugs do not alter *E*_*MSH*_ of *Mtb* during growth in culture medium, they specifically induce intramycobacterial oxidative *E*_*MSH*_ to exert efficient mycobactericidal activity during infection [14]. Collectively, these findings underscore the importance of MSH-redox homeostatic mechanism(s) in protecting mycobacteria against antibiotics. Contrary to known anti-TB drugs, treatment with ATD-3169 produces ROS inside mycobacteria and induces an intrabacterial oxidative shift in *E*_*MSH*_ of *Mtb* in culture medium *in vitro*. To decisively show that ATD-3169 functions by disturbing mycothiol redox balance, we assessed the effect of ATD-3169 on an *Mtb* H37Rv strain deficient in mycothiol biosynthesis (*Mtb*Δ*mshA*). The *Mtb*Δ*mshA* strain does not produce mycothiol and showed sensitivity to oxidants [38,39]. We found that the *Mtb*Δ*mshA* strain was 10 times more sensitive to ATD-3169 as compared to wt *Mtb* H37Rv (MIC 0.3125 μM) (Table 1). Complementation of *Mtb*Δ*mshA* with an integrated copy of wt *mshA* restored the sensitivity to *Mtb* H37Rv levels. These results suggest that the mycothiol system is one of the main intrinsic mechanisms for protection against ATD-3169 in *Mtb*.

Similar to ATD-3169, another redox-cycling drug, clofazimine (CFZ), has been shown to induce mycobacterial killing by generating ROS and inducing an oxidative shift in *E*_*MSH*_ during growth in culture medium [14]. Therefore, we checked if the CFZ and ATD-3169 combination acts synergistically on *Mtb*. First the MICs of CFZ and ATD-3169 were determined independently using an AB assay and found to be consistent with previously reported MIC values for CFZ (Table 2). Second, using a checkerboard assay (see Experimental procedures), compound interactions were determined by growing *Mtb* strains in sub-MIC concentrations of CFZ and sub-MIC fractions of ATD-3169. The ΣFIC (fractional inhibitory concentration) for each combination was calculated and given in Table 2. The combination of CFZ and ATD-3169 gave a ΣFIC of ≤0.5 against the drug-sensitive laboratory strain (H37Rv) and drug-resistant clinical strains (Jal 2287 and MYC 431), indicating that both drugs act synergistically and therefore affect similar aspects of mycobacterial redox physiology. As a control, we performed a checkerboard assay using an isoniazid (INH) and ATD-3169 combination and found no synergy (data not shown), thereby confirming that the antimycobacterial activity of ATD-3169 is linked to ROS generation and MSH redox imbalance. Taken together, our data for the first time revealed that *Mtb* is uniquely sensitive to elevation in endogenous ROS levels, and suggest selective targeting of mycobacterial redox homeostasis by producing excessive oxidative stress within *Mtb* cells as a potential new intervention strategy against TB.

### ATD-3169 modulates expression of genes associated with the antioxidant system, DNA repair, central metabolism, and cell wall lipid biosynthesis

**Fig. 5:**
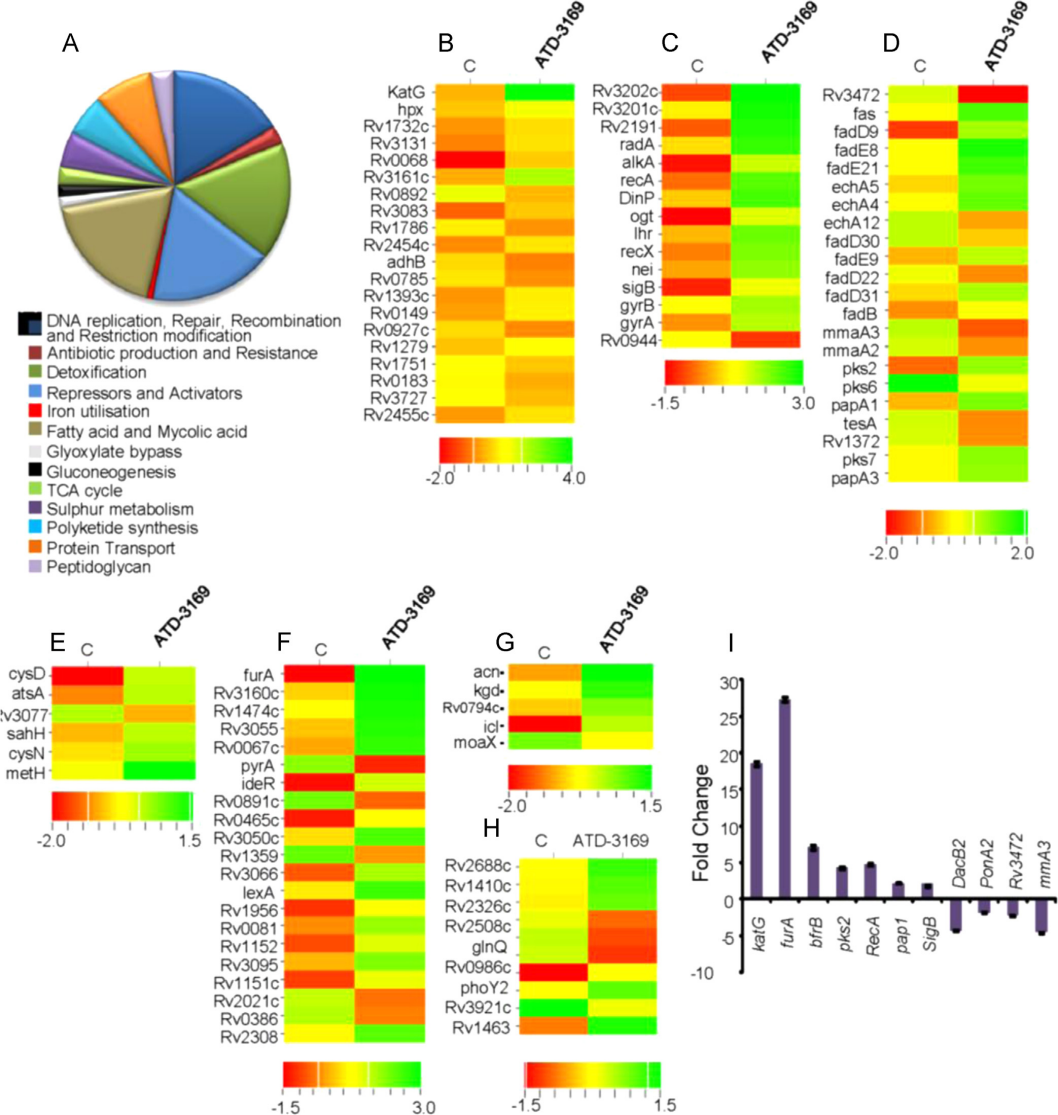
Global changes in the expression of *Mtb* H37Rv genes in response to ATD-3169. *Mtb* H37Rv was grown to an O.D_600 nm_ of 0.4 and treated with 30 μM ATD-3169 for 4 h at 37 °C. Total RNA was isolated and subjected to microarray analysis as noted under Materials and methods. (A) Pie chart corresponding to differentially expressed genes in response to ATD-3169, functionally classified in 13 classes based on the annotation given in the tubercuList database. Differentially regulated genes were shown in the heat maps. (B) Detoxification, miscellaneous oxidoreductases, and oxygenases, (C) DNA replication, repair, recombination, and restriction/modification, (D) fatty acid catabolism and lipid anabolism, (E) sulfur metabolism, (F) transcriptional regulators, (G) TCA cycle and glyoxylate cycle genes, and (H) drug efflux pumps. (I) qRT-PCR validation of a select number of genes differentially regulated in the microarray data. Data shown are the representative of at least two difference experiments performed in triplicate. Error bars represents standard deviations from the mean.

To further understand the mechanism of ATD-3169 action, we performed microarray analysis of *Mtb* H37Rv exposed to 30 µM ATD-3169 for 4 h (see SI Experimental procedures). A transcriptional response showed differential regulation of ~500 genes in response to ATD-3169 (2-fold cutoff, *P*≤0.05, Table S2). Since ROS generally damage DNA, proteins, and lipids, a transcriptional response to ATD-3169 is largely composed of genes involved in DNA repair, cell wall lipid biosynthesis, iron homeostasis, sulfur metabolism, redox-active proteins, citric acid cycle proteins, and transcriptional regulators (Fig. 5A and Table S2). Given that ATD-3169 induced substantial oxidative stress, expression of *katG* was highly upregulated (~35-fold) on treatment. Additionally, several genes involved in the oxidation–reduction reactions such as *hpx* (heme haloperoxidase), *Rv1786* (ferredoxin), oxidoreductases (*Rv0068, Rv0149, Rv0183*), and monooxygenases (Rv0892, Rv3083, Rv1393c) were influenced by ATD-3169 (Fig. 5B). Because DNA is one of the sensitive targets of ROS, we expected to see many genes involved in DNA metabolism to be differentially expressed in response to ATD-3169. Consistent with this, among the highly upregulated genes were those which encode for helicases (*uvrC, uvrD)*, DNA repair and recombination (*recA*, *radA*, *dinP*, *alkB*, *nei*, etc.), DNA supercoiling (*gyrA*, *gyrB*), SOS response (*lexA*), etc. (Fig. 5C). The upregulation of several genes involved in cell wall lipid/polyketide biosynthesis (*mma3, mma2, pks2, pks7, papA1, papA3*, etc.) and fatty acid metabolism (*fas, fadD9, fadD22, fadD31*, etc.) indicates damage of the cell wall lipid architecture by ATD-3169 (Fig. 5D). Since ATD-3169 inhibits growth of *Mtb* by perturbing mycothiol redox homeostasis, we reasoned that pathways involved in the biosynthesis of intracellular thiols might be affected by this compound. In agreement with this, genes involved in the biosynthesis of reduced sulfur metabolites such as cysteine and methionine [*metH, cysN, cysD, sahH*, etc.] and the prominent sulfur-containing cell wall glycolipid, sulfolipid-1 (*pks 2, papA1*) were induced on treatment (Fig. 5E).

Because iron homeostasis is intrinsically coupled to ROS-mediated lethality [12,40], genes involved in iron storage (*bfrB*, *Rv2455c*) and iron regulation (*ideR*, *furA*) were highly induced in our expression data (Fig. 5F). The induction of iron-repressor and iron-storage genes indicates the increase in free iron content on treatment with ATD-3169, suggesting a possible leaching of iron by endogenous O_2_^−•^ from iron-binding proteins such as Fe–S cluster proteins. In line with this, we observed overexpression of *csd,* which encodes for a cysteine desulfurase involved in the repair of oxidatively damaged Fe–S clusters [41]. One of the unified mechanisms by which ROS inhibit growth of prokaryotic and eukaryotic organisms is by releasing iron from the citric acid cycle enzyme like [Fe–S]-dependent dehydratase, aconitase [28]. Interestingly, we found that ATD-3169 induced expression of multiple genes which encode for citric acid cycle enzymes including Fe–S containing aconitase (*acn*), 2-oxoglutarate dehydrogenase (*kgd*), and dihydrolipoamide dehydrogenase (*Rv0794c*), indicating a direct role of endogenous ROS generated by ATD-3169 in inactivating these mycobacterial enzymes (Fig. 5G). Additionally, genes involved in glyoxylate cycle (isocitrate lyase; *icl*) and gluconeogenesis (phosphoenolpyruvate carboxykinase; *pck*), which counterbalance oxidative stress by increasing the yield of reducing power in mycobacteria [42,43], were found to be upregulated by ATD-3169 (Fig. 5G). These results indicate a functional linkage between energy metabolism and intracellular redox balance, and suggest a major role of central metabolism in increasing the efficiency of *Mtb* to tolerate oxidative stress.

Increased expression of multiple drug efflux pumps provides a critical defense against redox-cycling drugs in *Escherichia coli* [44]. Similarly, induction of genes encoding efflux pumps, including *Rv2688c, Rv1410c, Rv2326c, phoY2*, etc., indicates that *Mtb* tries to reduce endogenous ROS levels by limiting intracellular accumulation of ATD-3169 (Fig. 5H). *Mtb׳s* adaptive response to oxidative stress is dependent on multiple transcriptional regulators and stress-dependent sigma factors [8]. A large number of gene regulators including *Rv3160c, Rv1474c, Rv3055, Rv0067c, Rv3050c, ideR, furA,* etc. were induced by ATD-3169 (Fig. 5F). Lastly, using quantitative reverse-transcriptase PCR (qRT-PCR), we verified the microarray results by monitoring the differential expression of a select number of *Mtb* genes in response to ATD-3169 (Fig. 5I; Table S2, SI). In sum, data generated from multiple techniques clearly show that the efficient antimycobacterial activity shown by the ATD-3169 is a consequence of increased intrabacterial oxidative stress and associated damage to cellular pathways involved in ROS detoxification, and repair of essential metabolites such as DNA, proteins, and lipid in *Mtb*.

## Discussion

Increased emergence of drug-resistant *Mtb* strains coupled with a meager number of new antibiotics in the pipeline has necessitated an urgent need to enhance our portfolio of anti-TB drugs. In this context, a paradigmatic approach would be to induce long-lasting and irreversible changes in cellular homeostatic mechanisms of *Mtb*. Several studies in humans, animal models, and macrophages clearly indicate that exogenous oxidants such as O_2_^−•^ produced by a phagocytic respiratory burst are critical components of host defense against bacterial infection [3–5]. Furthermore, owing to exceptionally higher rate constants (10^6^ M^−1^, s^−1^) for O_2_^−•^-mediated inactivation of essential metabolic enzymes such as dehydratase and mononuclear enzymes, a modest increase in endogenous O_2_^−•^ levels elicits substantial enzymatic and growth defects in bacteria [45]. Despite the remarkable antimicrobial potency of exogenous oxidants, *Mtb* seems to express several mechanisms specifically directed to dissipate host-generated oxidative stress for persistence *in vivo*. While *Mtb׳s* ability to resist host-generated redox stress is widely studied, to the best of our knowledge how mycobacteria respond to a specific increase in endogenous O_2_^−•^ level has not been characterized to date. Consequently, we have developed a series of cell-permeable redox-cycling drugs which generate ROS, specifically O_2_^−•^ via undergoing bioreductive activation inside mycobacteria. Because O_2_^−•^ is a charged species at physiological pH (p*K*_a_=4.8), it cannot penetrate cellular membranes [27]. Keeping this in mind, ATD compounds were specifically designed to generate O_2_^−•^ inside a mycobacterial cell. The specific detection of O_2_^−•^ in the cytoplasm of mycobacteria validates our chemical synthesis approach and indicates a mechanism involving abstraction of electrons from low-potential metal centers (Fe–S clusters), respiratory quinones, and flavins followed by univalent reduction of O_2_ to generate O_2_^−•^ by ROS generators. Our results demonstrating exceptional sensitivity of *Mtb* (including MDR/XDR strains) to compounds that generate endogenous ROS, along with the reported resistance of *Mtb* toward exogenous oxidants, revealed crucial differences in defense mechanisms exploited by *Mtb* to tolerate ROS produced inside or outside the bacterial cell. Such disparate responses to ROS were not observed in other bacterial systems. For example, while under normal respiratory conditions basal scavenging enzymes (e.g., Sod, Kat) are sufficient to protect *E. coli* from endogenous O_2_^−•^ and H_2_O_2_, any abnormal increase in endogenous ROS levels by exogenous oxidative stress agents or redox-cycling drugs is rapidly sensed by OxyR and SoxRS systems, respectively, to mount an effective antioxidant response [46,47]. Interestingly, major antioxidant enzymatic systems in *Mtb* are either secretory (SodA, KatG) or located in the periplasm (SodC), indicating that the pathogen is evolutionarily equipped to neutralize extracellular O_2_^−•^ derived from phagocytes during infection. The above findings, along with the lack of a functional cytosolic redox-sensing system such as OxyR or SoxR, indicate that *Mtb* is genetically programmed to divert the majority of cellular antioxidant machinery to mitigate exogenous ROS and to maintain cytoplasmic redox balance. While important in the natural context of infection, these studies indicate that *Mtb* might be vulnerable to redox-cycling drugs that permeate inside bacteria to specifically elevate endogenous ROS levels and perturb redox homeostasis. Consistent with this, virulent H37Rv and MDR/XDR strains have shown exceptional sensitivity to an increase in endogenous ROS/RNS by redox-cycling drugs such as nitroimidazopyrans, CFZ, and vitamin C [12,27,44]. In contrast, other bacterial species (including *Msm*) known to contain nonsecretory forms of antioxidant enzymes and capable of inducing an OxyR-type protective oxidative stress response were comparatively more tolerant to killing by nitroimidazopyrans, vitamin C, and ATD compounds (Table 1) [35,48].

The production of millimolar concentrations of cytoplasmic redox buffer, MSH, is the main mechanism by which mycobacteria maintain a reduced cytosolic state [49]. We reasoned that if ATD-3169 specifically elevates endogenous ROS levels, then one would expect to observe perturbation of mycothiol redox homeostasis in *Mtb*. Using a novel mycothiol biosensor [14], we showed that ATD-3169 induces a rapid and long-lasting oxidative shift in *E*_*MSH*_ of *Mtb*, indicating impairment of MSH-controlled reductive capabilities of *Mtb* by ATD-3169. A similar oxidative shift in *E*_*MSH*_ was induced in MDR/XDR strains, raising an interesting possibility of targeting mycothiol redox pathway to impact survival of both drug-sensitive and drug-tolerant *Mtb* strains. The importance of the mycothiol pathway in tolerating endogenous ROS is further revealed by our findings showing an exceptionally high sensitivity of *MtbΔmshA* toward ATD-3169. The reported higher antioxidant capacity of *Msm* (*E*_*MSH*_=-300 mV) as compared to *Mtb* (*E*_*MSH*_=−270 mV) may have partly contributed to differential susceptibility of these strains toward ATD-3169 in our study [14]. Finally, our microarray data validate our estimates that ATD-3169 induces endogenous oxidative stress and perturbs mycobacterial redox metabolism. Similar to other redox-cycling drugs, ATD-3169 appears to function by disrupting iron homeostasis. Induction of genes encoding the Fe–S cluster containing TCA cycle enzymes, iron-storage proteins, Fe–S cluster repair proteins, and iron repressors clearly indicates elevated iron levels due to damage of Fe–S clusters by ATD-3169. Because iron is the coreactant in the Fenton reaction, which increased the chances of DNA damage, a large number of DNA repair genes were upregulated. Interestingly, we also observed activation of the gluconeogenesis pathway and drug-efflux pumps in response to ATD-3169. Since NADPH depletion is a most likely a consequence of redox cycling, it is possible that induction of genes involved in gluconeogenesis may be a mechanism to restore cellular NADPH levels. Similarly, upregulation of drug-efflux pumps is one of the predominant strategies exploited by bacteria to excrete redox-cycling drugs. Of note, the drug-efflux pumps induced by ATD-3169 were similarly regulated in response to a macrophage environment, anti-TB drugs, and redox agents, indicating their broader role in detoxification and maintaining cellular homeostasis [50,51]. While induction of many oxidative stress responsive genes was clearly evident, expression of *sodA*, ERG, and MSH biosynthetic genes was not affected on ATD-3169 treatment. Although unexpected, other expression studies have shown that many antioxidant systems including SOD are constitutively overexpressed in *Mtb* and that their expression remained uninfluenced in response to an oxidative or nitrosative insult [34,52]. Alternatively, it is likely that some posttranscriptional mechanisms are involved in regulating the levels of these antioxidants in *Mtb*. For example, levels of SodA are directly modulated by the activity of SecA2 secretion system in *Mtb* [53]. Similarly levels of ERG can be regulated by the transport system(s) involved in its secretion [54]. Lastly, changes in metabolite levels can alter levels of MSH. Since cysteine and methionine biosynthetic genes are differentially expressed in response to ATD-3169, MSH levels can be regulated by changes in the flux of cysteine or methionine on oxidative stress. Interestingly, homologues of many ATD-3169-inducible genes were similarly regulated by O_2_^−•^ and H_2_O_2_ in a SoxR- and/or OxyR-dependent manner in other bacteria, indicating the existence of novel redox-sensitive transcription factors in *Mtb*. In this context, some of the members of the *Mtb whiB* family (Fe–S cluster containing redox-sensitive transcription factors) appear to modulate the oxidative stress response in *Mtb* [55,56].

In summary, our biochemical, genetic, and expression experiments indicate that ATD-3169 enhances intramycobacterial ROS levels that trigger bactericidal activity by weakening redox homeostasis in *Mtb*. Due to the pressing need for developing novel interventional methodologies to counter *Mtb*, the effects of directly increasing endogenous ROS, in real time, on the intracellular redox potential of *Mtb* and its effects on mycobacterial physiology and survival were studied. We find that *Mtb* is uniquely sensitive to perturbation in redox homeostasis when it encounters elevated ROS within cells. *Mtb* secretes large amounts of major antioxidant enzymes such as catalase and superoxide dismutase to mitigate ROS encountered inside host phagocytes. Impaired survival of *Mtb* strains lacking either secretory antioxidants or major cell wall lipids in animal models of experimental tuberculosis further emphasizes the importance of mechanisms to alleviate toxicity associated with exogenous oxidants generated during infection. The evolutionary role of effectively countering exogenous ROS appears to have compromised *Mtb׳*s ability to overcome endogenous ROS. We anticipate that our findings will play an important role in high content screening of small-molecule inhibitors of intrabacterial redox homeostasis and in delineating novel redox pathways involved in persistence, drug resistance, and pathogenesis of *Mtb.* Increased sensitivity displayed by *Mtb* toward elevated endogenous ROS may be relevant to other intracellular pathogens (e.g., *Salmonella* and *Meningococcus*) which mainly exploit secretory antioxidant enzymes/systems to effectively divert exogenous superoxide during infection. Thus, our findings may have relevance to several intracellular pathogens causing chronic and relapsing infections where persistence and drug tolerance pose challenges for treatment.

## Figures and Tables

**Table 1:** Minimal inhibitory concentrations (MICs) of ROS generators against diverse bacterial species.

Strain	ATD-3167	ATD-3169	ATD-4053	ATD-4054	ATD-4064	ATD-4110
*Staphylococcus aureus*	>100 µM	>100 µM	>100 µM	>100 µM	>100 µM	>100 µM
*E. coli*	>100 µM	>100 µM	>100 µM	>100 µM	>100 µM	>100 µM
*Salmonella typhimurium*	>100 µM	>100 µM	>100 µM	>100 µM	>100 µM	>100 µM
*Klebsiella pneumoniae*	>100 µM	>100 µM	>100 µM	>100 µM	>100 µM	>100 µM
*Pseudomonas aeruginosa*	>100 µM	>100 µM	>100 µM	>100 µM	>100 µM	>100 µM
*Msm* mc^2^155	200 µM	50 µM	>200 µM	>200 µM	200 µM	>200 µM
*H37Rv*	100 µM	3.125 µM	100 µM	>50 µM	12.5 µM	12.5 µM
MDR (Jal 2287)	50 µM	3.125 µM	25 µM	>50 µM	6.25 µM	6.25 µM
XDR (Myc-431)	100 µM	6.25 µM	50 µM	>50 µM	25 µM	25 µM
MDR (Jal 2261)	ND	3.125 µM	ND	ND	ND	ND
MDR (Jal 1934)	ND	3.125 µM	ND	ND	ND	ND
MDR (BND320)	ND	3.125 µM	ND	ND	ND	ND
*M. bovis* BCG	ND	3.125 µM	ND	ND	ND	ND
*H37Rv* Δ*MshA*	ND	0.3125 µM	ND	ND	ND	ND
*H37Rv* Δ*MshA* comp	ND	3.125 µM	ND	ND	ND	ND
*Msm*Δ*mshA*	ND	25 µM	ND	ND	ND	ND
*Msm*Δ*mshD*	ND	25 µM	ND	ND	ND	ND

MICs of ATD compounds against a range of Gram-positive and Gram-negative bacteria were measured using the microbroth dilution technique as recommended by National Committee for Clinical Laboratory Standards. MIC, minimum inhibitory concentration; ND, not determined.

**Table 2:** Data showing synergy between ATD-3169 and clofazimine (CFZ).

Strain	Drug combination	MIC (µM for 3169, µg/ml for clofazimine)		FIC	FICI
		Alone	Combination		
H37Rv	3169	3.125	0.7	0.23	0.47
	Clofazimine	0.25	0.0625	0.24	
Jal2287 (MDR)	3169	3.125	0.7	0.23	0.478
	Clofazimine	0.125	o.031	0.248	
Myc 431 (XDR)	3169	6.25	1.5	0.24	0.49
	Clofazimine	0.5	0.125	0.25	

Influence of ATD-3169 and CFZ combination against drug-sensitive (H37Rv), multidrug resistant (MDR: Jal 2287), and extensively drug-resistant (XDR: Myc 431) strains of *Mtb* was examined using a checkerboard assay.
