# Supplementary material for: Mycobacterium tuberculosis has diminished capacity to counteract redox stress induced by elevated levels of endogenous superoxide
Source: Free Radic Biol Med. 2015 Jul;84:344–54. doi: 10.1016/j.freeradbiomed.2015.03.008 (PMC4459714; doi:10.1016/j.freeradbiomed.2015.03.008)
Supplement: Supplementary file 1 — Supplementary Material [file mmc1.pdf]

Supplemental Information

For

Mycobacterium tuberculosis has diminished capacity to counteract redox stress induced by elevated levels of endogenous superoxide

Priyanka Tyagi[1, 2#], Allimuthu T. Dharmaraja[3#], Ashima Bhaskar[1], Harinath Chakrapani[3*], and Amit Singh[1*]

[1]Department of Microbiology and Cell Biology, Centre for Infectious Disease Research (CIDR), Indian Institute of Science, Bangalore-12, India, [2]International Centre for Genetic Engineering and Biotechnology, New Delhi-67, India, [3]Department of Chemistry, Indian Institute of Science Education and Research, Pune- 08, India

To whom correspondence should be addressed: Harinath Chakrapani (Assistant Professor), Department of Chemistry, IISER, Pune-08, email: harinath@iiserpune.ac.in, Tel: 91-20-2590-8090 and Amit Singh (Assistant Professor), Department of Microbiology and Cell Biology, IISc, Bangalore-12, email: asingh@mcbl.iisc.ernet.in, Tel: 91-80-2293-3275

Experimental procedures

All reactions were conducted under a nitrogen atmosphere. All the chemicals were purchased from commercial sources and used as received unless stated otherwise. Dichloromethane (DCM), toluene and tetrahydrofuran (THF) for reaction were used as dried, and petroleum ether and ethyl acetate (EtOAc) for chromatography were used as received from commercial sources. Column chromatography was performed on Merck silica gel (100–200 mesh). ^{1}H and ^{13}C spectra were recorded on JEOL 400 MHz (or 100 MHz for ^{13}C) spectrometers using either residual solvent signals as an internal reference ($CDCl_3$ δ_H, 7.24 ppm, δ_C 77.1 ppm) or an internal tetramethylsilane ($\delta_H = 0.00$, $\delta_C = 0.0$). The chemical shifts (δ) are reported in ppm and coupling constants (*J*) in Hz. The following abbreviations are used: m (multiplet), s (singlet), br s (broad singlet), d (doublet), t (triplet) dd (doublet of doublet) and dt (doublet of triplet). High-resolution mass spectra were obtained from HRMS-ESI-Q-Time of Flight LC/MS. FT-IR spectra were obtained using NICOLET 6700 FT-IR spectrophotometer as KBr disc and reported in cm^{-1}. Melting point was measured using a VEEGO melting point apparatus. All melting points were measured in open glass capillary and values are uncorrected. High performance liquid chromatography (HPLC) was performed on Agilent model with Zorbax SB C-18 reverse phase column (250 × 4.6 mm, 5μm). Fluorimetric, luminometric and spectrophotometric measurements were performed using Thermo Scientific Varioscan microwell plate reader.

Bacterial strains and growth conditions. The bacterial species and strains used in this study were *Escherichia coli, Salmonella typhimurium, Klebsiella pneumonia, Pseudomonas aeruginosa, Mycobacterium smegmatis* (*Msm*) mc^{2}155, *MsmΔmshA*, *MsmΔmshD*, *Mycobacterium bovis* BCG (Pasteur), *Mycobacterium tuberculosis* (*Mtb*) strains *H37Rv*, *H37RvΔMshA*, *H37RvΔMshA* comp and the drug resistant field isolates Jal 1934, Jal 2287, Jal 2261, BND 320 and MYC 431 (kind gift from Dr. Kanury V.S. Rao, ICGEB, New Delhi). *Mtb* strains were cultures in liquid Middlebrook 7H9 broth (Difco) or solid 7H11 (Difco) supplemented with 0.05% Tween 80, 0.1% glycerol, 1X Albumin Dextrose Saline (ADS) and appropriate drugs kanamycin (25 μg/ml) or hygromycin (50 μg/ml).

Superoxide detection by luminol Assay:[1]. 5-Amino-2,3-dihydro-1,4-phthalazinedione solution (Luminol, 4 mM) was prepared in 30 mM aqueous sodium hydroxide and stored under ice. To a microwell plate, a stock solution of the compound (2 μL of 1 mM) was added to phosphate buffer (100 mM pH 8.0, 193 μL) followed by luminol (5 μL, final 100 μ M in 200 μ L). The resulting mixture was incubated at 37ºC for 25 min during which the luminescence was measured using a microtiter plate reader. Hypoxanthine (2 μL, 1 mM) and xanthine oxidase (2 μL of 0.02 U/mL) were used as a positive control. Superoxide dismutase (SOD from Bovine erythrocytes, sigma), 10 U/mL was prepared in 1 mM phosphate buffer (1 mM in phosphate buffer pH 7.8. The complete quenching of luminescence was observed during the addition of SOD.

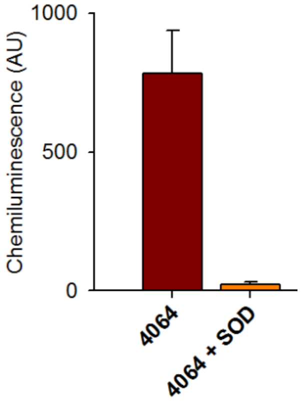

Figure S1. Luminol assay for detection of superoxide generated from **4064**, with and without SOD.

Intracellular superoxide detection by dihydroethidine (DHE) assay. [2-4]. *Mycobacterium smegmatis* ($MC^2$155) was cultured in 5 mL of middle brook M7H9 medium (M7H9, with 10% albumin-dextrose-saline (ADS) supplement) at 37ºC for 24 h. The cultured bacteria were centrifuged to aspirate out the medium and re-suspended to an $O.D_{600\ nm}$ of 1.0 with fresh M7H9 medium. This bacterial solution was incubated with 250 μM of dihydroethidine (DHE) for 30 min in dark by covering the falcon tube in an aluminum foil. The suspension was centrifuged to aspirate out any excess DHE in the medium. The collected bacterial pellet was re-suspended with fresh M7H9 medium. This DHE pre-treated bacterial solution was incubated with 250 μM of ATD-3169 for 30 min in dark by covering the falcon tube in an aluminum foil. The suspension was centrifuged to aspirate out

any excess of ATD-3169 in the medium. The collected bacterial pellet was re-suspended with acetonitrile and the cells were lysed using a probe sonicator for 3 min on ice. The cell lysate was then removed by centrifugation and the supernatant acetonitrile was separated and stored at -20 ºC before injecting in HPLC. The HPLC method used was as described in the methods sections.[2-4]

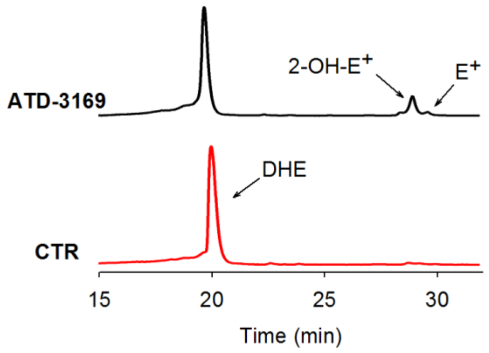

Figure S2. HPLC traces of assay for intracellular $O_2^{-\bullet}$production using a hydroethidine (DHE) assay in *Mycobacterium smegmatis* (*Wt-Msm*). Incubation with ATD-3169 (250 µM) was for 30 min and DHE levels indicate unoxidized dye while 2-OH-E^+ formed is an indicator for $O_2^{-\bullet}$ production and E^+ is indicative of increase in oxidative species.

Extracellular hydrogen peroxide estimation. [5]. *Mycobacterium smegmatis* ($MC^2$155) was cultured in 5 mL of middle brook 7H9 medium (7H9, with 10% albumin-dextrose-saline (ADS) supplement) at 37ºC for 24 h. The cultured bacteria were centrifuged to aspirate out the medium and re-suspended to an O.D of 0.5 with fresh 7H9 medium. A stock solution of the compound in DMSO (0.5%) was added so that the final concentration was 50 µ M. After incubation for 1 h to the bacterial suspension (divided in several 50 µ L portions in a 96-well microplate), 50 µ L of a premixed solution of 10-acetyl-3,7- dihydroxyphenoxazine or Amplex Red® (prepared by following manufacturer's protocol from Invitrogen) was added and incubated at RT for 25 min before measuring the fluorescence using a microtiter plate reader (excitation 550 nm; emission 590 nm). A calibration curve with known concentrations of H_2O_2 was generated (data not shown) in 7H9 medium and was used to quantify H_2O_2produced during incubation of test analytes. Data presented are an average of two independent experiments, each carried out in triplicate.

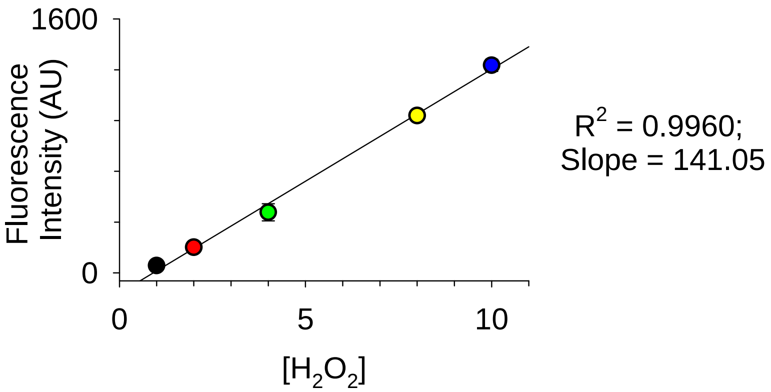

Figure S3. Calibration curve generated for H_2O_2 of concentration ranging from 1-10 μM in middle brook 7H9 medium.

Synthesis and characterization data. General procedure for Synthesis of 2,3-dihydronaphthoquinones, their NMR characterization is given below. Compounds ATD-3167 [6], ATD-4064 [7] and ATD-4110 [7] have been previously reported and analytical data that we collected were consistent with the reported values.

RNA isolation for microarray and quantitative RT-PCR. 30 ml of *Mtb* culture was grown in 7H9 broth (Difco) supplemented with ADS till it reaches to an O.D $_{600nm}$ of 0.4 in roller culture bottles at 2 rpm. Three times the volume of buffer containing 5 M Guanidinium thiocyanate, 0.5% sarcosyl, 0.5% Tween-80, 1% β-mercaptoethanol was added to the culture mixed quickly and cells were harvested by centrifugation. Pellet was resuspended in 2 ml fast prep reagent (MP-Bio) and was lysed in "bead beater vials" containing zarconia beads. Debris was spin down and clean supernatant was transferred into fresh tubes. Chloroform was mixed in supernatant and centrifuged for phase separation. An equal volume of isopropanol was added to aqueous phase and incubated at -80ºC. RNA was purified using an RNeasy kit (Qiagen) in accordance with the manufacturer's instructions.

DNA microarrays were provided by University of Delhi South Campus MicroArray Centre (UDSCMAC). RNA amplification, CDNA labeling, microarray hybridization, scanning and data analysis were performed at the UDSCMAC as described [8]. Slides were scanned on a microarray scanner (Agilent Technologies) and the resulting TIFF images were analyzed using the GeneSpring software. Data transformation and normalization was carried out by taking threshold 1, logbase 2, Technology: Agilent.SingleColor.34585 and Normalization: shift to 50 percentile. Results were analysed in MeV with Significance Analysis of Microarrays considered significant at p<=0.05. The normalized data from the microarray gene expression experiment has been submitted to NCBI's Gene Expression Omnibus (GEO, http://www.ncbi.nlm.nih.gov/geo/) and can be queried via GEO series accession number GSE61618.

Quantitative RT-PCR (qRT-PCR) was performed using gene-specific primers (Sigma Aldrich) and iScript™ One-Step RT-PCR Kit with SYBR® Green (Bio-Rad) on a CFX96 Touch™ Real-Time PCR Detection System as described previously [9]. Sequences of primers used in the study are described in supplementary information (Table S3). Each experiment was performed in triplicate. Expression was normalized using 16S rRNA expression as an internal housekeeping control.

General procedure for Synthesis of 2,3-dihydronaphthoquinones. To a 25 mL round bottom flask a solution of compound (0.5 mmol) in tetrahydrofuran (THF, 6 mL) was purged with nitrogen gas for 10 min, Palladium on charcoal (Pd/C, 1 mol %) was added and stirred under H_2 atmosphere for 30 min at room temperature. Upon complete consumption of starting material (by TLC analysis), the reaction mixture was filtered through a celite bed and the bed was washed with THF (5 mL). The resulting filtrate was evaporated under reduced pressure and washed with *n*-pentane (3 × 3 mL) to obtain pure product. Following the general procedure compounds ATD-3167, ATD-3169, ATD-4053, ATD-4064, and ATD-4054 were synthesized.

5-Hydroxy-1,2,3,4,4a,9a-hexahydro-1,4-methano-9,10-anthraquinone (ATD-3169). Starting from **2** (120 mg, 0.50 mmol), **ATD-3169** was isolated as a yellow solid (102 mg, 91%): mp 139 – 141 °C; FT-IR (υ_{max}, cm^{-1}): 3450, 2943, 2937, 1667, 1633, 1578, 1456, 1416, 1355, 1304, 1263, 1230, 1168, 1067; ^1H NMR (400 MHz, $CDCl_3$): δ 12.79 (s, 1H), 7.61-7.68 (m, 2H), 7.26 (dd, *J* = 0.9, 8.0 Hz, 1H), 3.21 (dd, *J* = 4.7, 10.8 Hz, 1H), 3.12 (dd, *J* = 4.8, 10.8 Hz, 1H), 3.01 (d, *J* = 13 Hz, 2H), 1.45-1.60 (m, 4H), 1.13-1.20 (m, 2H); ^{13}C NMR (100 MHz, $CDCl_3$): δ 206.4, 198.4, 162.1, 137.5, 135.5, 124.1, 118.3, 118.2, 50.3, 50.0, 43.9, 43.8, 39.3, 4.9, 24.8; HRMS (ESI-TOF): calcd. for $C_{15}H_{14}O_3$ $[M+Na]^+$: 265.0841; Found: 265.0845.

5,8-dihydroxy-1,2,3,4,4a,9a-hexahydro-1,4-methanoanthracene-9,10-dione (ATD-4053). Starting from **3** (150 mg, 0.59 mmol), **ATD-4053**was isolated as a yellow crystalline solid (132 mg, 87%): mp 138 – 140 °C; FT-IR (υ_{max}, cm^{-1}): 3440, 3088, 2950, 2876, 1620, 1451, 1360, 1310, 1205, 1072, 1023, 934; ^1H NMR (400 MHz, $CDCl_3$): δ 12.78 (s, 2H), 7.24 (s,2H), 3.13 (s, 2H), 2.98 (s, 2H), 1.50 (m, 4H), 1.17 (m, 2H); ^{13}C NMR (100 MHz,

$CDCl_3$): δ 205.0, 156.0, 129.1, 114.9, 49.7, 44.0, 39.3, 24.8; HRMS (ESI-TOF) for [$C_{15}H_{14}O_4+H^+$]: calcd., 259.0970. Found: 259.0972.

5,8-dihydroxy-1,2,3,4,4a,9a-hexahydro-1,4-ethanoanthracene-9,10-dione (ATD-4054). Starting from **5** (150 mg, 0.56 mmol), **ATD-4054**was isolated as a orange red crystalline solid (143 mg, 95%): mp 152 – 154 °C; FT-IR (υ_{max}, cm^{-1}): 3441, 3080, 2935, 2863, 1604, 1488, 1453, 1370, 1314, 1211, 1029, 951; ^{1}H NMR (400 MHz, $CDCl_3$): 12.75 (d, *J* = 1.6 Hz, 2H), 7.26 (d, *J* = 1.4 Hz, 2H), 3.10 (s, 2H), 2.36 (s, 2H), 1.73 (m, 4H), 1.43 (m, 4H); ^{13}C NMR (100 MHz, $CDCl_3$): δ 205.0, 155.8, 128.8, 114.5, 48.0, 30.3, 25.7, 21.9; HRMS (ESI-TOF) for [$C_{16}H_{16}O_4+H^+$]: calcd., 273.1121. Found: 273.1125.

Table S1. Synthesis of ATD-series of compounds

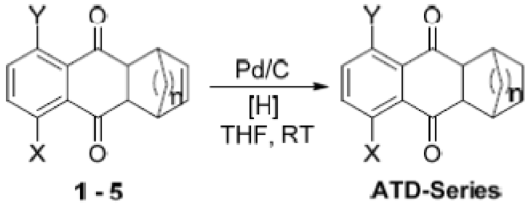

Entry	X	Y	n	Reactant	Product	Yield, %
1	H	H	1	**1**	**ATD-3167**	79
2	OH	H	1	**2**	**ATD-3169**	91
3	OH	OH	1	**3**	**ATD-4053**	87
4	OH	H	2	**4**	**ATD-4064**	88
5	OH	OH	2	**5**	**ATD-4054**	95

NMR Spectra: NMR spectra of ATD compounds”

^{1}H NMR spectra of **ATD-3169**:

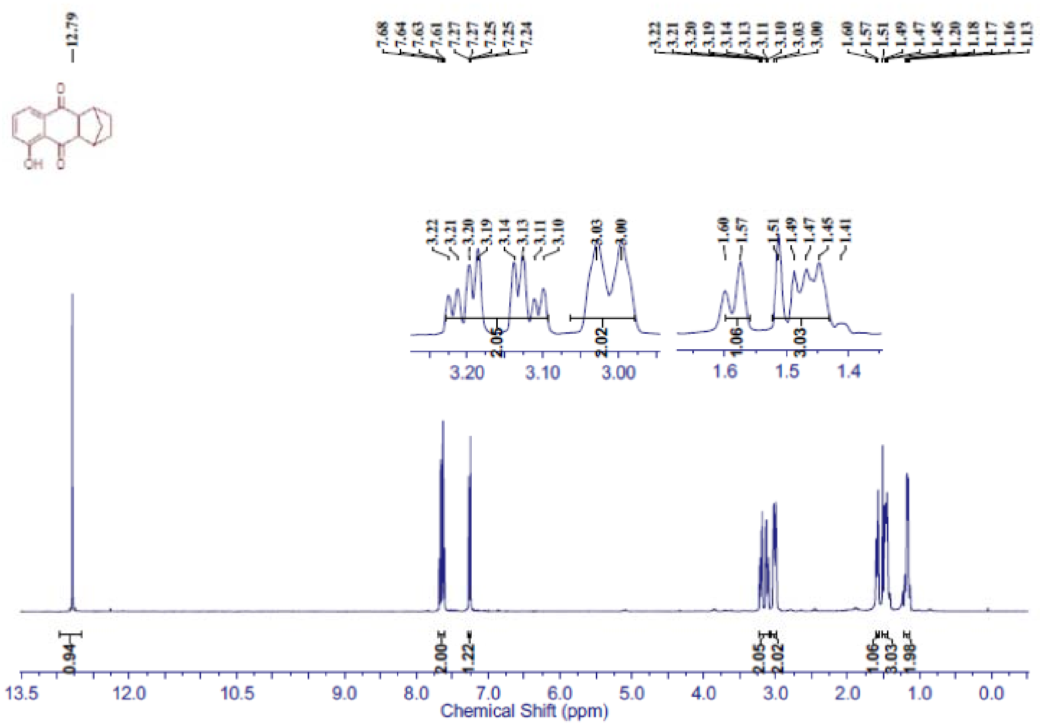

^{13}C NMR spectra of **ATD-3169**:

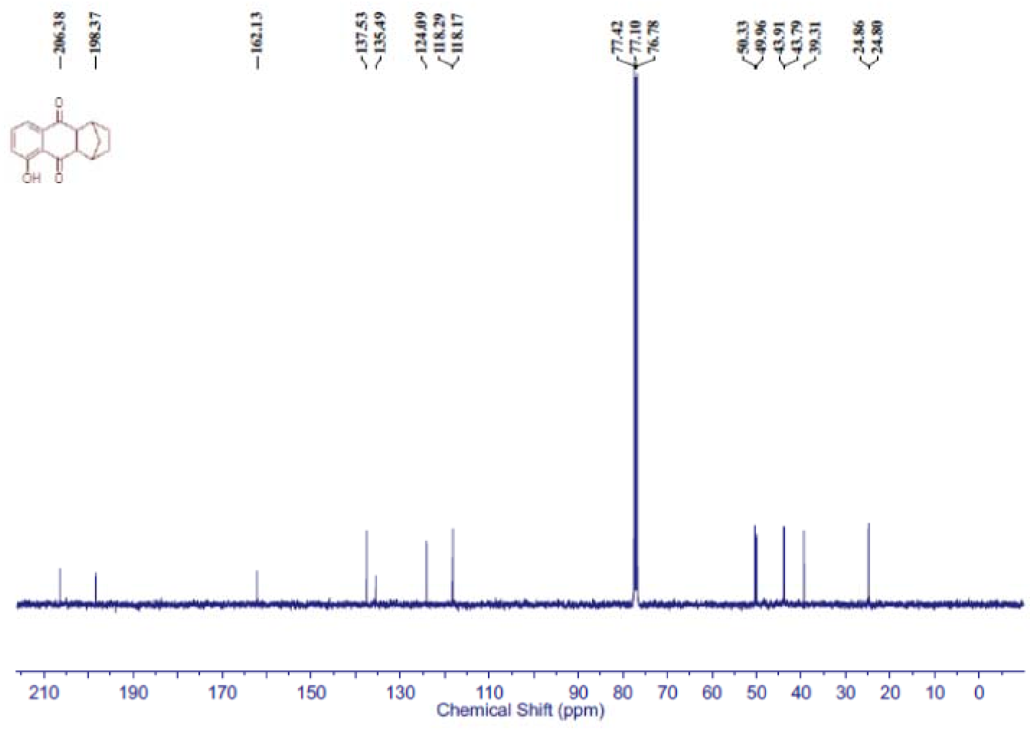

^{1}H NMR spectra of **ATD-4053**:

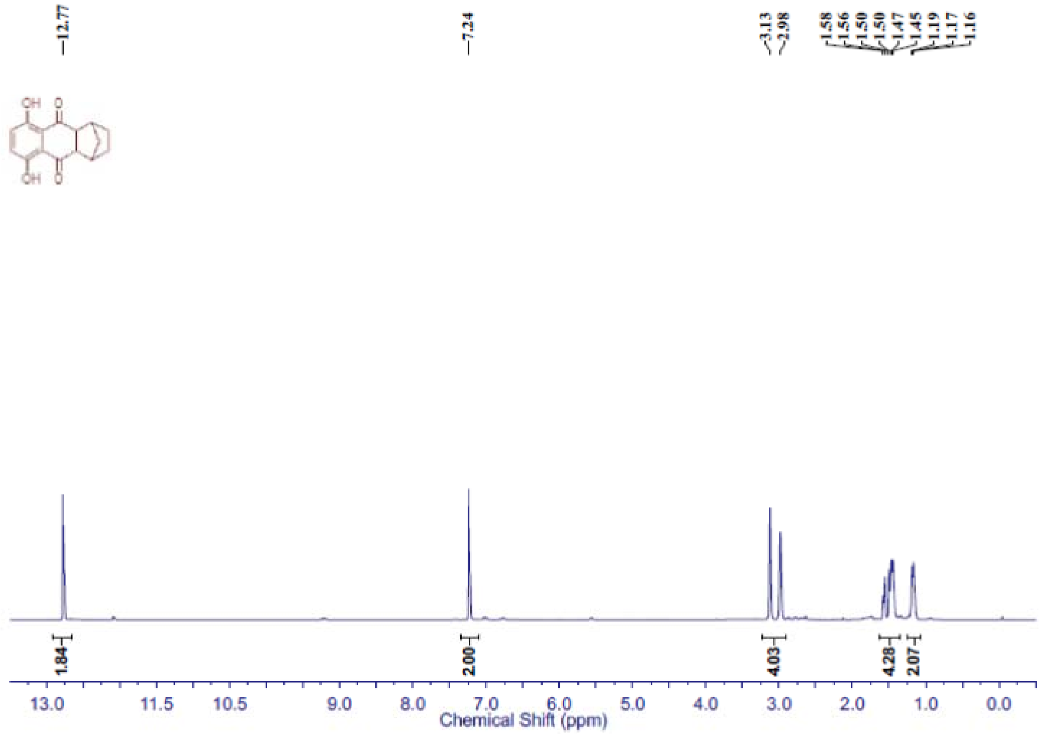

^{13}C NMR spectra of **ATD-4053**:

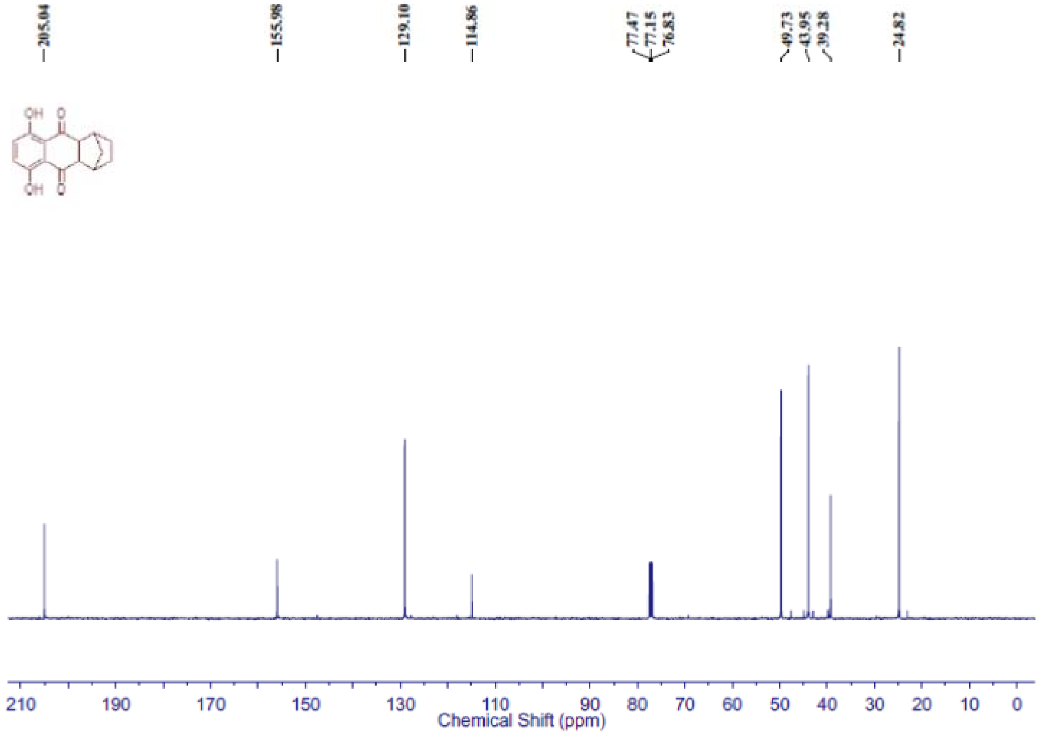

^{1}H NMR spectra of **ATD-4054**:

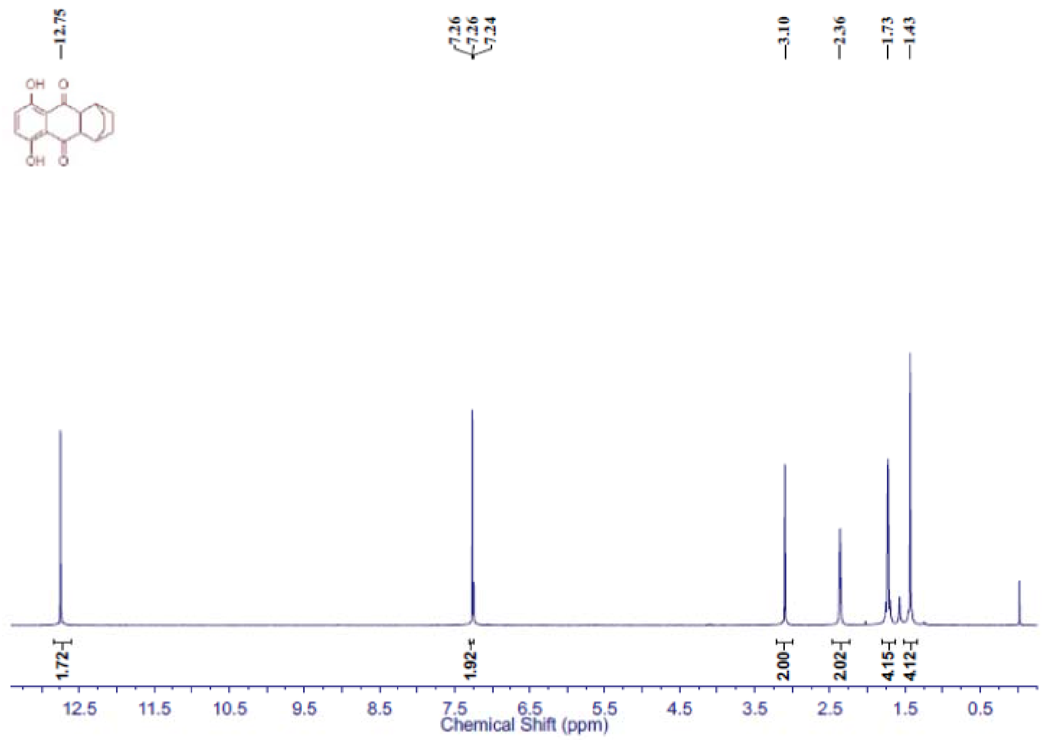

^{13}C NMR spectra of **ATD-4054**:

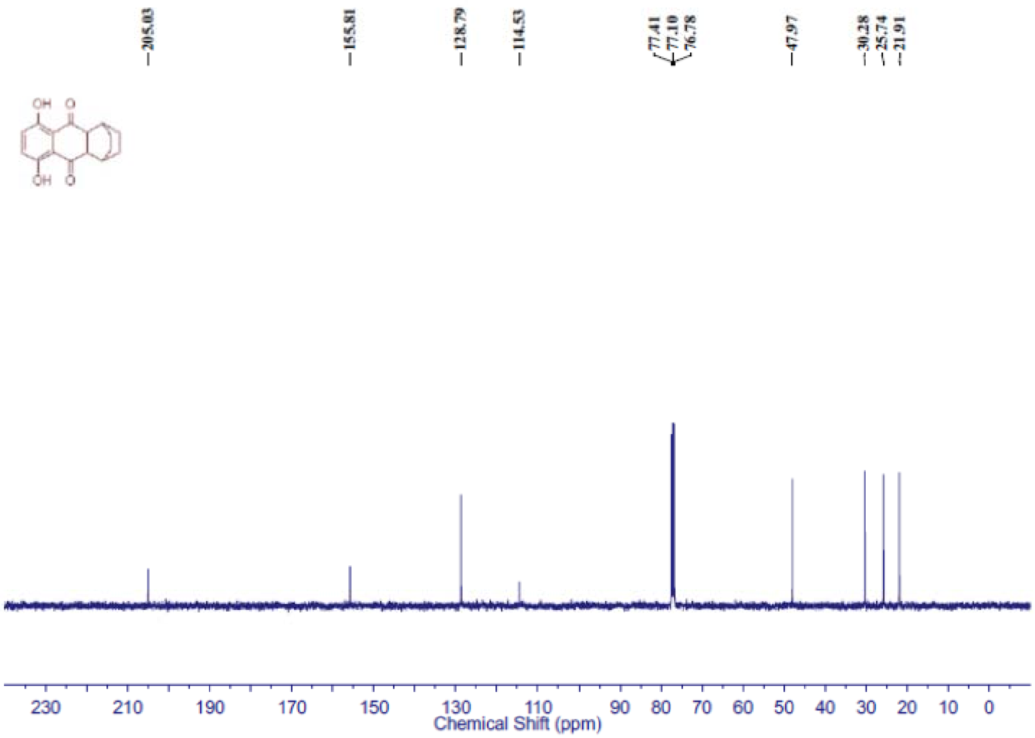

Table S2: Microarray profile of *Mtb* H37Rv treated with ATD-3169 (see enclosed SI excel spread sheet).

Table S3: Oligonucleotides used to perform qRT-PCR analysis. Sequences of primers utilized in validating the expression of genes identified by microarrays.

Oligonucleotide	Oligonucleotide Sequences (5'-3')
RT *furA* F	CCCGACGTATCCCGGCAAGC
RT furA R	GGGTGCCTCGCCAACAGCAC
RT *katG*F	CCCATGGCGCCGGCCCG
RT *katG* R	CGATGCCGCTGGTGATCGCG
RT *bfrA*F	CAGACCCCGCGAGGCACTGG
RT *bfrA* R	CAACCCGCACCAGGGTTGCC
RT Rv3472 F	CGACGGCTGGGTGATCCGGG
RT Rv3472 R	GCCGGCGGCAGTGGCCAG
RT *mmA3* F	CTCGACGTGGGCTGCGGCTG
RT *mmA3* R	GGTGCGAGCGGTTGGTGTCG
RT *RecA*F	CGGCCCGGAGTCGTCGGGT
RT *RecA* R	CGGCTGGCTGACCAGCAGCG
RT *SigB* F	ATGGCCGATGCACCCACAAG
RT *SigB* R	CTTGGCCAGTTCGACTTCAC
RT *pks2* F	AAGTGTCTCCGAGGTGTATG
RT *pks2* R	CGAGTGAAGTGCAGATTACG
RT *papA1* F	CCCGGCGTGTTAGTTTCGTC
RT *papA1* R	ACACCTCAACGGACGCAATC
RT *ponA2* F	GCAGCCATTCTCCCTGGTTG
RT *ponA2* R	CGCCACTTCCCAGACCTTTG
RT *dacB2* F	AACTGCGTCGGCGTCAAACC
RT *dacB2* F	GGCTTTGGCGTTCATCTTGG

References:

[1] Trung Pham, H.; Marquetty, C.; Pasquier, C.; Hakim, J. Luminol assay for microdetermination of superoxide dismutase activity: Its application to human fetal blood. *Anal. Biochem.* **142:**467-472; 1984.

[2] Georgiou, C. D.; Papapostolou, I.; Grintzalis, K. Superoxide radical detection in cells, tissues, organisms (animals, plants, insects, microorganisms) and soils. *Nat. Protoc.,* **3:**1679-1692; 2008.

[3] Zielonka, J.; Vasquez-Vivar, J.; Kalyanaraman, B. Detection of 2-hydroxyethidium in cellular systems: A unique marker product of superoxide and hydroethidine. *Nat. Protoc.,* **3:**8-21; 2008.

[4] Zhao, H.; Joseph, J.; Fales, H. M.; Sokoloski, E. A.; Levine, R. L.; Vasquez-Vivar, J.; Kalyanaraman, B. Detection and characterization of the product of hydroethidine and intracellular superoxide by HPLC and limitations of fluorescence. *Proc. Natl. Acad. Sci.,* **102:**5727-5732; 2005.

[5] Zhou, M.; Diwu, Z.; Panchuk-Voloshina, N.; Haugland, R. P. A Stable Nonfluorescent Derivative of Resorufin for the Fluorometric Determination of Trace Hydrogen Peroxide: Applications in Detecting the Activity of Phagocyte NADPH Oxidase and Other Oxidases. *Anal. Biochem.,* **253:**162-168; 1997.

[6] Menter, J. M.; Filipescu, N. Photochemical detection of intramolecular energy transfer between nonconjugated chromophores in rigid model compounds. *J. Chem. Soc. (B)***:**464-468; 1970.

[7] Dharmaraja, A. T.; Alvala, M.; Sriram, D.; Yogeeswari, P.; Chakrapani, H. Design, synthesis and evaluation of small molecule reactive oxygen species generators as selective Mycobacterium tuberculosis inhibitors. *Chem. Commun.,* **48:**10325-10327; 2012.

[8] Venkataraman, B.; Vasudevan, M.; Gupta, A. A new microarray platform for whole-genome expression profiling of Mycobacterium tuberculosis. *J Microbiol Methods* **97:**34-43; 2014.

[9] Chawla, M.; Parikh, P.; Saxena, A.; Munshi, M.; Mehta, M.; Mai, D.; Srivastava, A. K.; Narasimhulu, K. V.; Redding, K. E.; Vashi, N.; Kumar, D.; Steyn, A. J.; Singh, A. Mycobacterium tuberculosis WhiB4 regulates oxidative stress response to modulate survival and dissemination in vivo. *Mol Microbiol* **85:**1148-1165; 2012.
